# Radiative analysis of luminescence in photoreactive systems: Application to photosensitizers for solar fuel production

**DOI:** 10.1371/journal.pone.0255002

**Published:** 2021-07-22

**Authors:** Caroline Supplis, Jérémi Dauchet, Victor Gattepaille, Fabrice Gros, Thomas Vourc’h, Jean-François Cornet

**Affiliations:** Université Clermont Auvergne, Clermont Auvergne INP, CNRS, Institut Pascal, F-63000 Clermont-Ferrand, France; College of Charleston, UNITED STATES

## Abstract

Most chemical reactions promoted by light and using a photosensitizer (a dye) are subject to the phenomenon of luminescence. Redistribution of light in all directions (isotropic luminescence emission) and in a new spectral range (luminescence emission spectrum) makes experimental and theoretical studies much more complex compared to a situation with a purely absorbing reaction volume. This has a significant impact on the engineering of photoreactors for industrial applications. Future developments associated with photoreactive system optimization are therefore extremely challenging, and require an in-depth description and quantitative analysis of luminescence. In this study, a radiative model describing the effect of luminescence radiation on the calculation of absorptance is presented and analyzed with the multiple inelastic-scattering approach, using Monte Carlo simulations. The formalism of successive orders of scattering expansion is used as a sophisticated analysis tool which provides, when combined with relevant physical approximations, convenient analytical approximate solutions. Its application to four photosensitizers that are representative of renewable hydrogen production *via* artificial photosynthesis indicates that luminescence has a significant impact on absorptance and on overall quantum yield estimation, with the contribution of multiple scattering and important spectral effects due to inelastic scattering. We show that luminescence cannot be totally neglected in that case, since photon absorption lies at the root of the chemical reaction. We propose two coupled simple and appropriate analytical approximations enabling the estimation of absorptance with a relative error below 6% in every tested situation: the zero-order scattering approximation and the gray single-scattering approximation. Finally, this theoretical approach is used to determine and discuss the overall quantum yield of a bio-inspired photoreactive system with Eosin Y as a photosensitizer, implemented in an experimental setup comprising a photoreactor dedicated to hydrogen production.

## 1 Introduction

Molecular homogeneous photoreactive systems (*i.e*. chemical molecules or clusters of molecules which can interact with light and then react) have many applications and significant industrial interest. We can cite first of all applications in industrial photochemistry using artificial light sources [1]. More recently, new solar applications for green photochemistry [2, 3], the production of renewable hydrogen from water [4–6] or carbon dioxyde photoreduction [7–9] involving artificial photosynthesis (solar fuels) are becoming increasingly important. This last application, which motivated the present work, is a considerable challenge in the 21^st^ century. Having efficient and inexpensive catalysts for the photolysis of water, associated with solar processes designed with high thermodynamic efficiency, is the guarantee of being able to easily store solar energy in chemical vectors.

In the quest for cheap catalyst development, molecular catalysts synthesized from relatively abundant elements on Earth (in contrast with particle catalysts based on doped-semiconductors, which are still more effective today [6, 10–12] but out of the scope of this article), are an attractive prospect for the future [13–15]. However, they must always be associated with one or more photosensitizers, which will initiate the reaction by elementary photon absorption leading to an excited state as a singlet or triplet. The use of these photosensitizers goes far beyond the framework of the production of solar energy vectors, since they are at the root of all the applications mentioned above.

One of the characteristic phenomena encountered in the use of photosensitizers is that electrons in the first excited state can spontaneously relax to the ground state by fluorescence (for a singlet) or phosphorescence (for a triplet) emission without a reactive event. These photoluminescence phenomena are both responsible for light emission in the volume, at the expense of photoreaction. We argue that photoluminescence requires a sound description and quantitative analysis when formulating the thermokinetic coupling law and performing the radiative balance in a photoreactor, for two reasons. First, photons emitted by luminescence at one location in the medium can potentially be absorbed at another location and participate in the reaction. Therefore, photoluminescence cannot be treated simply as a loss for the reaction; it must be addressed in the description of photon transport within the photoreactor. Thus, the considerations of luminescence in transmittance and absorptance calculations or measurements may be of crucial importance as regards the above-mentioned photochemical and artificial photosynthesis applications. Second, photoluminescence is responsible for light emission in all directions (isotropic emission) and with a spectral distribution (emission spectrum) that is different from that of the incident radiation. The radiative balance in the photoreactor then becomes extremely challenging without appropriate modeling and analysis tools.

In more detail, from the engineering point of view, and considering that photoreactive processes are controlled at different scales by radiative transfer (or photon transport) [16], an understanding of the photon absorption process (leading to a reactive event) is essential to the radiative analysis, and thus to the optimization of any photoreactive process at the application scale. It is therefore necessary to gain further insights into this physical phenomenon, which can be quantified properly by determining the local A(r) and spatial (mean) 〈A〉 volumetric rates of photons absorbed (MVRPA), where we have introduced for convenience the bracket notation $\langle •\rangle =\frac{1}{V}{\;\int \int \int \;}_{V}•\;dV$. This quantification also serves as a basis for the formulation of coupling laws between the photon absorption rate and the local chemical reaction rate *r*_*i*_(**r**). The physical or engineering treatment of any photoreactive system relies on a local thermokinetic coupling involving an overall quantum yield *φ* with the general form [16, 17]:

$$\begin{array}{c} r_{i}\left(r\right)=\varphi A\left(r\right) \end{array}$$ (1)

Local coupling is required because the local volumetric rate of photons absorbed (LVRPA) field A(r) is highly heterogeneous when most of the incident radiation is absorbed in the vessel, which is the aim of photoreactive processes. Nevertheless, if the quantum yield *φ* does not depend on the radiation field, which is often the case for molecular photoreactive systems as studied in this article, averaging these two rates at the spatial scale of the enclosure (the scale of physical observable and measured quantities) is straightforward and leads to a linear thermokinetic coupling law [18, 19]:

$$\begin{array}{c} \left\langle r_{i}\right\rangle =\varphi \left\langle A\right\rangle \end{array}$$ (2)

In these situations, the kinetic and thermodynamic formulations of a knowledge model for any photoreactor geometry are fully provided by a thorough evaluation of the mean spatial volumetric rate of radiant energy absorbed 〈A〉 [16, 19, 20]. This description of luminescence has two important consequences in the thermokinetic coupling law (Eq 2):

- Luminescence cannot be considered as a loss in the thermokinetic coupling. Thus the luminescence quantum yield is not included in the overall quantum yield *φ*,
- Luminescence is included in the radiative model. It enables us to account for the multiple absorption and emission of luminescent radiation when estimating 〈A〉.

Even though the present study is of great interest for all applications using homogeneous (molecular) photoreactive systems, here we focused on four standard photosensitizers. They were chosen because they are widely used for the production of solar fuels, in association with a suitable catalyst and a sacrificial electron donor. The luminescent molecules studied in this paper are either fluorescent molecules such as Eosin Y [21–24], derived from triazatriangulenium (TATA^+^) [25] and Rhodamine B [26, 27] or phosphorescent molecules such as tris(2,2’-bipyridine)ruthenium ($\text{Ru}{\left[\text{Bpy}\right]}_{3}^{2+}$) [28, 29]. They were treated using a unified approach, requiring only basic information (experimental data or DFT calculations, for example) such as spectral extinction coefficient, luminescence emission spectrum and luminescence quantum yield.

Regarding the application, we considered a homogeneous reaction medium with luminescent species implemented in a photoreactor modeled as a slab whose radiative configuration and boundary conditions are detailed in section 2.1. In particular, heterogeneous photoreactive systems with particles leading to elastic scattering are not discussed in this article. First we will focus on situations where the luminescent photosensitizer is the only species that interacts with radiation—there is no other absorbing species—and results will subsequently be extended to configurations where the catalyst also absorbs radiation. Extension is quite straightforward and the conclusions are not modified, since absorption by catalyst is not desirable (it does not lead to reaction) and is therefore low when working with efficient molecular photoreactive systems.

The corresponding steady-state Radiative Transfer Equation (RTE) will be presented in section 2.2 and is rigorously solved using the Monte Carlo method. Efforts are then essentially focused on the analysis of luminescence effects on MVRPA, and as a result on the construction of relevant analytical approximate solutions. The collision term that describes luminescence in the radiative transfer equation can either be interpreted as an emission source in the volume [30, 31], or as an inelastic and isotropic scattering phenomenon [32–34]. The second approach enables an intuitive understanding of the photon transport physics and was chosen here. Moreover, we took the decision to describe the MVRPA solution as successive orders of scattering for several reasons: i) it provides intuitive physical pictures when describing the absorption of luminescence radiation, ii) it is a common method used in radiative analysis to develop analytical approximate solutions [35], and iii) it is convenient to study the luminescence effect on 〈A〉, as will be presented in section 3.

The overall effect of luminescence on absorptance is quantified, indicating that luminescence can hardly be neglected in applications concerning solar fuel production. We therefore analyzed the respective influence of two characteristics of luminescence: multiple scattering and inelasticity. For this purpose, the weight of the successive scattering orders was first analyzed, and then the effect of inelasticity in scattering was quantified by comparing the reference results with those obtained thanks to equivalent gray or elastic scattering models. From these developments important conclusions decribing the impact of luminescence on MVRPA quantification are given, and rules of thumb for reasoning from well-defined optical thicknesses are proposed for any application using photosensitizers. Various useful analytical approximations for MVRPA assessment, also valid for any photosensitized reactive process in the absence of particles, are fully developed and compared to rigorous calculations in section 4 in order to discuss their validity in detail.

Finally, in section 5 the implementation of the proposed method will make it possible to revisit results already published regarding the use of a bio-inspired catalyst for the production of H_2_ in photoreactors, where the fluorescence of Eosin Y was neglected [18]. This will enable us to accurately estimate the overall quantum yields *φ* and thus to refine the performances achievable by this kind of photoreactive system.

## 2 Radiative model

### 2.1 Radiative configuration

**Fig 1:**
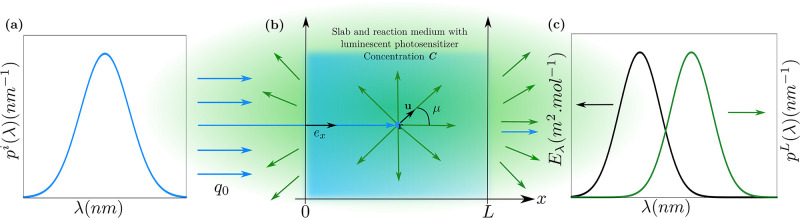
Radiative configuration. **(a)** Incident-normal collimated radiation at *x* = 0 with spectrum *p*^*i*^(λ) and surface flux density *q*_0_. **(b)** Slab of thickness *L* containing a luminescent photosensitizer solution at concentration *C*. The phase space (**r**, **u**), with **u** the propagation direction, can be reduced to (*x*, *μ*) in such a one-dimensional slab. In this case, the isotropic distribution $p\left(u\right)=\frac{1}{4\pi }$ becomes $p\left(\mu \right)=\frac{1}{2}$ as $\int_{4\pi }du\;\frac{1}{4\pi }=\int_{0}^{2\pi }\frac{d\varphi }{2\pi }\int_{0}^{\pi }d\theta \;\frac{1}{2}\;sin\theta =\int_{-1}^{1}d\mu \;p\left(\mu \right)$ with *μ* = cos *θ* [35]. **(c)** Examples of molar extinction cross-section *E*_λ_ and luminescence spectra *p*^*L*^(λ) of the photosensitizer.

Photon transport was modeled in a one dimensional slab of thickness *L* with incident-normal collimated radiation on the left-hand side (see Fig 1). The incident monochromatic photon flux density inside the medium, at *x* = 0, is *q*_0,λ_ = *q*_0_ *p*^*i*^(λ), where $q_{0}=\int_{0}^{+\infty }d\lambda \;q_{0,\lambda }$ is the total photon flux density and *p*^*i*^(λ) is the incident spectral probability density function, hereafter called *incident spectrum*: $\int_{0}^{+\infty }d\lambda \;p^{i}\left(\lambda \right)=1$. The interfaces at both sides of the slab are assumed to be transparent in order to analyze the roles of luminescence and separate them from the impact of reflection-refraction at the boundaries.

The slab is filled with a homogeneous photocatalytic system solution including luminescent photosensitizer at concentration *C* (there is no particle leading to elastic scattering in such systems). In the present configuration the photosensitizer is the only molecule that interacts with radiation in the spectral range of interest (UV-visible). Some catalysts also absorb radiation and we will show in section 5 that the extension to this situation is straightforward.

Luminescence can either be interpreted as an emission source in the volume or as an inelastic scattering phenomenon, as will be discussed in Section 2.2. In this work, the luminescence of photosensitizers is approached from the multiple inelastic-scattering point of view, because of the intuitive physical pictures it provides when describing the absorption of luminescence radiation. There is therefore an important distinction between 1) extinction cross-section *E*_λ_, which characterizes all interactions with radiation, 2) scattering cross-section *E*_*S*,λ_, which characterizes interactions leading to luminescence emission only and 3) absorption cross-section *E*_*A*,λ_, which characterizes interactions leading to other phenomena, including photocatalytic reactions (and other relaxation phenomena), with *E*_λ_ = *E*_*S*,λ_ + *E*_*A*,λ_. The luminescence quantum yield Φ_λ_ is the proportion of interactions that leads to luminescence emission, and therefore Φ_λ_ = *E*_*S*,λ_/*E*_λ_ [36–38]. From the photon transport point of view, Φ_λ_ is the single-scattering albedo: if a photon interacts with a photosensitizer molecule, it is scattered with probability Φ_λ_ or absorbed with probability (1 − Φ_λ_). In the following, the radiative properties of photosensitizers are provided by the molar extinction cross-section *E*_λ_ and luminescence quantum yield Φ_λ_, since absorption and scattering cross-sections are easily deduced from these quantities: *E*_*A*,λ_ = (1 − Φ_λ_)*E*_λ_ and *E*_*S*,λ_ = Φ_λ_ *E*_λ_. In the same way, the extinction coefficient of the solution is *k*_λ_ = *CE*_λ_, the absorption coefficient is (1 − Φ_λ_)*k*_λ_ and the scattering coefficient is Φ_λ_ *k*_λ_. Scattering events redistribute propagation directions according to isotropic phase function [38] and wavelengths according to the luminescence spectral probability density function *p*^*L*^(λ|λ′), hereafter called *luminescence spectrum*: $\int_{0}^{+\infty }d\lambda \;p^{L}\left(\lambda |\lambda^{\prime }\right)=1$. The luminescence spectrum is the third radiative property of the photosensitizer, together with *E*_λ_ and Φ_λ_, that may vary depending on the photocatalytic system. In the following, Kasha’s rule is assumed, and therefore the luminescence quantum yield Φ and spectrum *p*^*L*^ do not depend on the excitation radiation wavelength: Φ_λ_ ≡ Φ and *p*^*L*^(λ|λ′) ≡ *p*^*L*^(λ) [39]. In other words, the spectral distribution of scattered photons is independent of the spectral distribution of photons incident on the molecule.

For the purpose of radiative analysis, we define the following dimensionless optical thicknesses:

- spectral extinction optical thickness
  $$\begin{array}{c} \tau_{\lambda }=CLE_{\lambda } \end{array}$$ (3)
- mean absorption optical thickness for incident radiation
  $$\begin{array}{c} \tau_{A}^{i}=\left(1-\Phi \right)CL{\bar{E}}^{i} \end{array}$$ (4)
  with the mean molar extinction cross-section over incident spectrum (gray approximation)
  $$\begin{array}{c} {\bar{E}}^{i}=\int_{0}^{+\infty }d\lambda \;p^{i}\left(\lambda \right)\;E_{\lambda } \end{array}$$ (5)
- mean scattering optical thickness for luminescence radiation
  $$\begin{array}{c} \tau_{S}^{L}=\Phi CL{\bar{E}}^{L} \end{array}$$ (6)
  with the mean molar extinction cross-section over luminescence spectrum (gray approximation)
  $$\begin{array}{c} {\bar{E}}^{L}=\int_{0}^{+\infty }d\lambda \;p^{L}\left(\lambda \right)\;E_{\lambda } \end{array}$$ (7)

The extinction optical thickness for luminescence radiation $\tau^{L}=CL{\bar{E}}^{L}$ will be also used to formulate approximations in Section 4.

In Section 3 $\tau_{A}^{i}$ and $\tau_{S}^{L}$ are used to characterize incident and luminescence photon transport, respectively. We choose to focus on the absorption of incident radiation because absorbing and converting incident radiation is the aim of photoreactive systems. On the other hand, we choose to focus on luminescence radiation scattering because the aim of this study is to analyze the impact of multiple inelastic scattering in such systems. However, note that it is straightforward to compute the scattering optical thickness for incident radiation $\tau_{S}^{i}=\Phi /\left(1-\Phi \right)\;\tau_{A}^{i}$ and the absorption optical thickness for luminescence radiation $\tau_{A}^{L}=\left(1-\Phi \right)/\Phi \;\tau_{S}^{L}$ from the data provided in Table 2, since the previous definitions of cross-sections lead to *τ* = *τ*_*S*_ + *τ*_*A*_ and Φ = *τ*_*S*_/*τ*.

### 2.2 Radiative transfer equation

The Radiative Transfer Equation (RTE) for a one-dimensional slab filled with a luminescent medium is

$$\begin{array}{c} \mu \frac{dI_{\lambda }\left(x,\mu \right)}{dx}=-k_{\lambda }I_{\lambda }\left(x,\mu \right)+\frac{1}{2}\int_{0}^{+\infty }d\lambda^{\prime }p^{L}\left(\lambda \right)\int_{-1}^{1}d\mu^{\prime }\Phi k_{\lambda^{\prime }}\;I_{\lambda^{\prime }}\left(x,\mu^{\prime }\right) \end{array}$$ (8)

where *I*_λ_ is the intensity, *μ* is the dot product between the propagation direction and **e**_*x*_ (see Fig 1). The extinction coefficient *k*_λ_, the single-scattering albedo Φ (the luminescent quantum yield) and the luminescence spectrum *p*^*L*^(λ) were defined in the previous paragraphs. The collision term in Eq 8 describes luminescence. When it is interpreted as an emission source in the volume, it can be read as follows:

-
$\int_{0}^{+\infty }d\lambda^{\prime }\int_{-1}^{1}d\mu^{\prime }k_{\lambda^{\prime }}\;I_{\lambda^{\prime }}\left(x,\mu^{\prime }\right)$ is the volumetric rate at which photons with all wavelength λ′, propagating in all directions *μ*′, are absorbed at location *x*,
- multiplying the above term by the luminescence quantum yield Φ leads to the rate of luminescence emission,
- this emission is spectrally distributed according to *p*^*L*^(λ), with isotropic distribution $\frac{1}{2}$ of emission directions *μ* (see Fig 1) [35].

When it is interpreted as an inelastic scattering phenomenon, it can be read as follows:

-
$\int_{-1}^{1}d\mu^{\prime }\Phi k_{\lambda^{\prime }}\;I_{\lambda^{\prime }}\left(x,\mu^{\prime }\right)$ is the volumetric rate at which photons with wavelength λ′, propagating in all directions *μ*′, are scattered at location *x*,
- multiplying the above term by the isotropic phase function (see Fig 1) $p\left(\mu |\mu^{\prime }\right)=\frac{1}{2}$ leads to the rate at which photons are scattered in direction *μ* (per unit *dμ*),
- *dλ*′*p*^*L*^(λ) is the probability that a photon with wavelength in *dλ*′ around λ′ is scattered with wavelength in *dλ* around λ,
- Integration over [0, + ∞[ sums the contributions of all wavelengths λ′.

The analysis in the article will be mainly based on this second interpretation.

For our configuration with normal collimated incident radiation, boundary conditions for *μ* > 0 are:

$$\begin{array}{ccc} I_{\lambda }\left(0,\mu =1\right) & = & q_{0,\lambda }=q_{0}\;p^{i}\left(\lambda \right) \end{array}$$ (9)

$$\begin{array}{ccc} I_{\lambda }\left(0,\mu \neq 1\right) & = & 0 \end{array}$$ (10)

### 2.3 Successive orders of scattering expansion

The analysis in this paper is based on expansion into successive orders of scattering (numerical solutions use the Monte Carlo method, see Section 2.5).

**Intensity** obeys the linear transport equation in Eq 8 and can therefore be expanded into successive orders of scattering [40–46]. The intensity *I* of the entire photon population is formulated as the sum of the intensities *I*^(*j*)^ corresponding to photons that have undergone *j* scattering events:

$$\begin{array}{c} I_{\lambda }\left(x,\mu \right)=\sum_{j=0}^{+\infty }I_{\lambda }^{\left(j\right)}\left(x,\mu \right) \end{array}$$ (11)

**Ballistic intensity** $I_{\lambda }^{\left(0\right)}\left(x,\mu \right)$ is due to photons that come directly from the incident-normal collimated source at the boundary *x* = 0, and is only attenuated within the medium:

$$\begin{array}{c} \mu \frac{dI_{\lambda }^{\left(0\right)}\left(x,\mu \right)}{dx}=-k_{\lambda }\;I_{\lambda }^{\left(0\right)}\left(x,\mu \right) \end{array}$$ (12)

with the boundary condition

$$\begin{array}{ccc} I_{\lambda }^{\left(0\right)}\left(0,\mu =1\right) & = & q_{0,\lambda } \end{array}$$ (13)

$$\begin{array}{ccc} I_{\lambda }^{\left(0\right)}\left(0,\mu \neq 1\right) & = & 0 \end{array}$$ (14)

**Order *j* intensity $I_{\lambda }^{\left(j\right)}\left(x,\mu \right)$** is due to photons that have undergone *j* scatterings before reaching phase-space location (*x*, *μ*): *I*^(1)^ accounts for photons that have undergone one scattering event, *I*^(2)^ accounts for photons that have undergone two scattering events, and so on. Each intensity *I*^(*j*>0)^ obeys a radiative transfer equation of its own, in which the source term corresponds to lower-order photons *I*^(*j*−1)^ that are scattered locally and move from population (*j* − 1) to population (*j*):

$$\begin{array}{c} \mu \frac{dI_{\lambda }^{\left(j\right)}\left(x,\mu \right)}{dx}=-k_{\lambda }I_{\lambda }^{\left(j\right)}\left(x,\mu \right)+\frac{1}{2}\int_{0}^{+\infty }d\lambda^{\prime }p^{L}\left(\lambda \right)\int_{-1}^{1}d\mu^{\prime }\Phi k_{\lambda^{\prime }}\;I_{\lambda^{\prime }}^{\left(j-1\right)}\left(x,\mu^{\prime }\right) \end{array}$$ (15)

with the boundary condition

$$\begin{array}{c} I_{\lambda }^{\left(j\right)}\left(0,\mu \right)=0 \end{array}$$ (16)

since no photon having undergone *j* > 0 scattering events is incident on the slab.

**Luminescence intensity $I_{\lambda }^{\left(S\right)}$** sums the contributions *j* > 0 of all scattered photons:

$$\begin{array}{c} I_{\lambda }^{\left(S\right)}=\sum_{j=1}^{+\infty }I_{\lambda }^{\left(j\right)} \end{array}$$ (17)

leading to (see Eq 11)

$$\begin{array}{c} I_{\lambda }\left(x,\mu \right)=I_{\lambda }^{\left(0\right)}\left(x,\mu \right)+I_{\lambda }^{\left(S\right)}\left(x,\mu \right) \end{array}$$ (18)

$I_{\lambda }^{\left(S\right)}$ obeys the following RTE, that is the sum of the RTEs in Eq 15 for all *j* > 0:

$$\begin{array}{ccc} \mu \frac{dI_{\lambda }^{\left(S\right)}\left(x,\mu \right)}{dx} & = & -k_{\lambda }I_{\lambda }^{\left(S\right)}\left(x,\mu \right)\\ & + & \frac{1}{2}\int_{0}^{+\infty }d\lambda^{\prime }p^{L}\left(\lambda \right)\int_{-1}^{1}d\mu^{\prime }\Phi k_{\lambda^{\prime }}\;I_{\lambda^{\prime }}^{\left(S\right)}\left(x,\mu^{\prime }\right)\\ & + & \frac{1}{2}\int_{0}^{+\infty }d\lambda^{\prime }p^{L}\left(\lambda \right)\int_{-1}^{1}d\mu^{\prime }\Phi k_{\lambda^{\prime }}\;I_{\lambda^{\prime }}^{\left(0\right)}\left(x,\mu^{\prime }\right) \end{array}$$ (19)

with the boundary condition

$$\begin{array}{c} I_{\lambda }^{\left(S\right)}\left(0,\mu \right)=0 \end{array}$$ (20)

This is the same RTE as that for intensity *I*_λ_ except that the source is not at the boundary because luminescence emission stimulated by ballistic photons *I*^(0)^ within the volume is the source of scattered photons, as formulated in the last term of Eq 19.

**A meaningful property** is due to the fact that the expansion has a zero-order closure: $I_{\lambda }^{\left(0\right)}$ has a closed-form expression, independent of the $I_{\lambda }^{\left(j>0\right)}$, and each higher order *j* > 0 is a function of $I_{\lambda }^{\left(j-1\right)}$ only. Therefore, truncating the expansion at order *q* means that the contribution of photons that have undergone more than *q* scattering events is neglected. As a result, the *q*-th order expansion in successive orders of scattering systematically underestimates intensity.

### 2.4 Radiative quantities of interest

**The Mean Volumetric Rate of Photons Absorbed** 〈A〉 (**MVRPA**) is the key radiative quantity in the study of photoreactive systems [16]. It is obtained by integrating the intensity *I* over directions, locations and wavelengths:

$$\begin{array}{c} {\left\langle A\right\rangle }^{\left(•\right)}=\frac{1}{L}\int_{0}^{+\infty }d\lambda \int_{0}^{L}dx\int_{-1}^{1}d\mu \;\left(1-\Phi \right)k_{\lambda }I_{\lambda }^{\left(•\right)}\left(x,\mu \right) \end{array}$$ (21)

where superscript ^(•)^ is dropped in the formulation of the total rate of absorption 〈A〉; otherwise, take • ≡ *j* for the rate ${\langle A\rangle }^{\left(j\right)}$ at which photons that have undergone *j* scattering events are absorbed, and • ≡ *S* for the rate ${\langle A\rangle }^{\left(S\right)}=\sum_{j=1}^{+\infty }{\langle A\rangle }^{\left(j\right)}$ at which scattered (*i.e*. luminescence) photons are absorbed:

$$\begin{array}{c} \left\langle A\right\rangle =\sum_{j=0}^{+\infty }{\left\langle A\right\rangle }^{\left(j\right)}={\left\langle A\right\rangle }^{\left(0\right)}+{\left\langle A\right\rangle }^{\left(S\right)} \end{array}$$ (22)

**Absorptance** $P_{A}$ is the key quantity in our radiative analysis. It can be interpreted as the proportion of photons that are absorbed in the medium, leading in our 1D cartesian geometry to:

$$\begin{array}{c} \left\langle A\right\rangle =\frac{q_{0}}{L}P_{A} \end{array}$$ (23)

The maximum photon absorption rate ${\langle A\rangle }_{max}$ is then obtained with $P_{A,max}$, which is lower than 1 if any scattering phenomenon exists in the medium. $P_{A,max}=1$ would be only achieved if ballistic and scattered radiation could be completely absorbed. We chose to base the radiative analysis in Section 3 mainly on absorptance rather than MVRPA, because it enables us to clearly distinguish the impact of:

- photon transport, which is contained in $P_{A}$ only and depends on optical thicknesses,
- dimension *L* of the system, which depends on the experimental setup or the engineering specifications when designing units for industrial-scale production (note that for a given dimension *L*, optical thickness can be controlled by adjusting the photosensitizer concentration *C*),
- incident photon flux *q*_0_, which depends on the source and fluctuates in solar conditions.

Substituting Eq 21 in Eq 23 leads to:

$$\begin{array}{c} P_{A}^{\left(•\right)}=\frac{1}{q_{0}}\int_{0}^{+\infty }d\lambda \int_{0}^{L}dx\int_{-1}^{1}d\mu \;\left(1-\Phi \right)k_{\lambda }I_{\lambda }^{\left(•\right)}\left(x,\mu \right) \end{array}$$ (24)

Overall, expansion into successive orders of scattering is:

$$\begin{array}{c} P_{A}=\sum_{j=0}^{+\infty }P_{A}^{\left(j\right)}=P_{A}^{\left(0\right)}+P_{A}^{\left(S\right)} \end{array}$$ (25)

**The distribution *P*^(*j*)^ of scattering orders** within the expansion indicates the respective weight of each scattering order:

$$\begin{array}{c} P^{\left(j\right)}=\frac{P_{A}^{\left(j\right)}}{P_{A}} \end{array}$$ (26)

The cumulative $P^{\left(j\leq q\right)}=\sum_{j=0}^{q}P^{\left(j\right)}$ is the proportion of absorption that is described by the *q*-th order expansion in successive orders of scattering. Alternatively, the complementary (tail) cumulative $P^{\left(j>q\right)}=\sum_{j=q+1}^{+\infty }P^{\left(j\right)}$ is the relative error on both absorptance $P_{A}$ and MVRPA 〈A〉 when the expansion is truncated at the *q*-th order. Of course, $\sum_{j=0}^{+\infty }P^{\left(j\right)}=1$ and *P*^(*j* > *q*)^ = 1 − *P*^(*j* ≤ *q*)^ according to Eq 25. In addition, the contribution of luminescence radiation is $P^{\left(S\right)}=P^{\left(j>0\right)}=P_{A}^{\left(S\right)}/P_{A}$, and we have *P*^(0)^ + *P*^(*S*)^ = 1. Note that *P*^(*j*)^ can also be interpreted as the proportion of photons absorbed after *j* scattering events with respect to all the absorbed photons, or as the probability that a photon is absorbed after *j* scattering events.

### 2.5 Monte Carlo algorithm

An analytical solution for the above multiple inelastic-scattering radiative model is not available. Our study will therefore use a Monte Carlo numerical solution, which has three main advantages: 1) the algorithms are intuitive and easily modified to evaluate physical hypotheses, as will be presented in Section 3.3; 2) it provides a reference solution including statistical error estimation and 3) even if the present work is conducted in a one-dimensional cartesian configuration, it is straightforward to extend implementation to complex geometry in subsequent studies [47].

The Monte Carlo algorithm presented below was used to estimate the absorptance $P{}_{A}$ and each scattering order $P_{A}^{\left(j\right)}$ for a given concentration *C*, slab thickness *L* and radiative properties Φ and *E*_λ_. It consists in sampling *N* independent realizations *w*_*i*_, *i* = 1, …*N* with the following sampling procedure:

- **Step 1**: Initialization of Monte Carlo weights *w*_*i*_ = 0 and $w_{i}^{\left(q\right)}=0$ for q∈N, scattering counter *j* = 0, emission location **r** = **0** and emission direction **u** = **e**_*x*_ (see Fig 1).
- **Step 2**: Wavelength λ is sampled according to the incident spectrum *p*^*i*^(λ), and the extinction coefficient *k*_λ_ = *CE*_λ_ is interpolated from spectral database.
- **Step 3**: A first extinction length *l* is sampled over [0, +∞[ according to the Bouguer extinction probability density function $p_{L}(l)=k_{\lambda }e^{-k_{\lambda }l}$ and the location is updated: **r** = **r** + *l* **u**.
- **while** 0 ≤ **r** · **e**_*x*_ ≤ *L* (location is inside the slab) **do**
  **Step 4**: Bernoulli test: uniform sampling of a realization *ξ* over [0, 1]
  - **if** *ξ* < Φ (scattering event) *then*
    **Step 4a.1**: Scattering counter is incremented: *j* = *j* + 1.
    **Step 4a.2**: Scattering direction **u** is sampled according to the isotropic phase function.
    **Step 4a.3**: Wavelength λ is sampled according to the luminescence spectrum *p*^*L*^(λ) and the extinction coefficient *k*_λ_ = *CE*_λ_ is interpolated from the spectral database.
    **Step 4a.4**: Extinction length *l* is sampled over [0, +∞[ according to the Bouguer extinction probability density function $p_{L}(l)=k_{\lambda }e^{-k_{\lambda }l}$ and the location is updated: **r** = **r** + *l* **u**.
    **end**
  - **Else**
    **Step 4b.1**: Path sampling is terminated due to absorption:
    • weights are computed:

$w_{i}^{\left(j\right)}=1$

    *w*_*i*_ = 1
    • go to the end of Monte Carlo realization.
    **end**
  **end**

Absorptance is estimated as:

$$\begin{array}{c} P_{A}≃\frac{1}{N}\sum_{i=1}^{N}w_{i} \end{array}$$ (27)

with standard error:

$$\begin{array}{c} \sigma \left(P_{A}\right)=\frac{1}{\sqrt{N-1}}\sqrt{\frac{1}{N}\sum_{i=1}^{N}{w_{i}}^{2}-(\frac{1}{N}\sum_{i=1}^{N}w_{i}{)}^{2}} \end{array}$$ (28)

In Tables 2 and 3, the number *N* of Monte Carlo realizations has been adjusted to obtain a relative uncertainty $\frac{\Delta P_{A}}{P_{A}}$ which is always smaller than 1%, with $\Delta P_{A}$ defined as the 95% confidence interval: $\Delta P_{A}=t_{0.05,N-1}\sigma \left(P_{A}\right)$, where *t*_0.05,*N*−1_ is the Student quantile (*t*_0.05,*N*−1_ ≃ 2 for a typical number *N* of Monte Carlo realizations). Scattering orders $P_{A}^{\left(j\right)}$ are obtained in the same way, but replacing *w*_*i*_ by $w_{i}^{\left(j\right)}$ in Eqs 27 and 28.

## 3 A potpourri of photosensitizers

**Fig 2:**
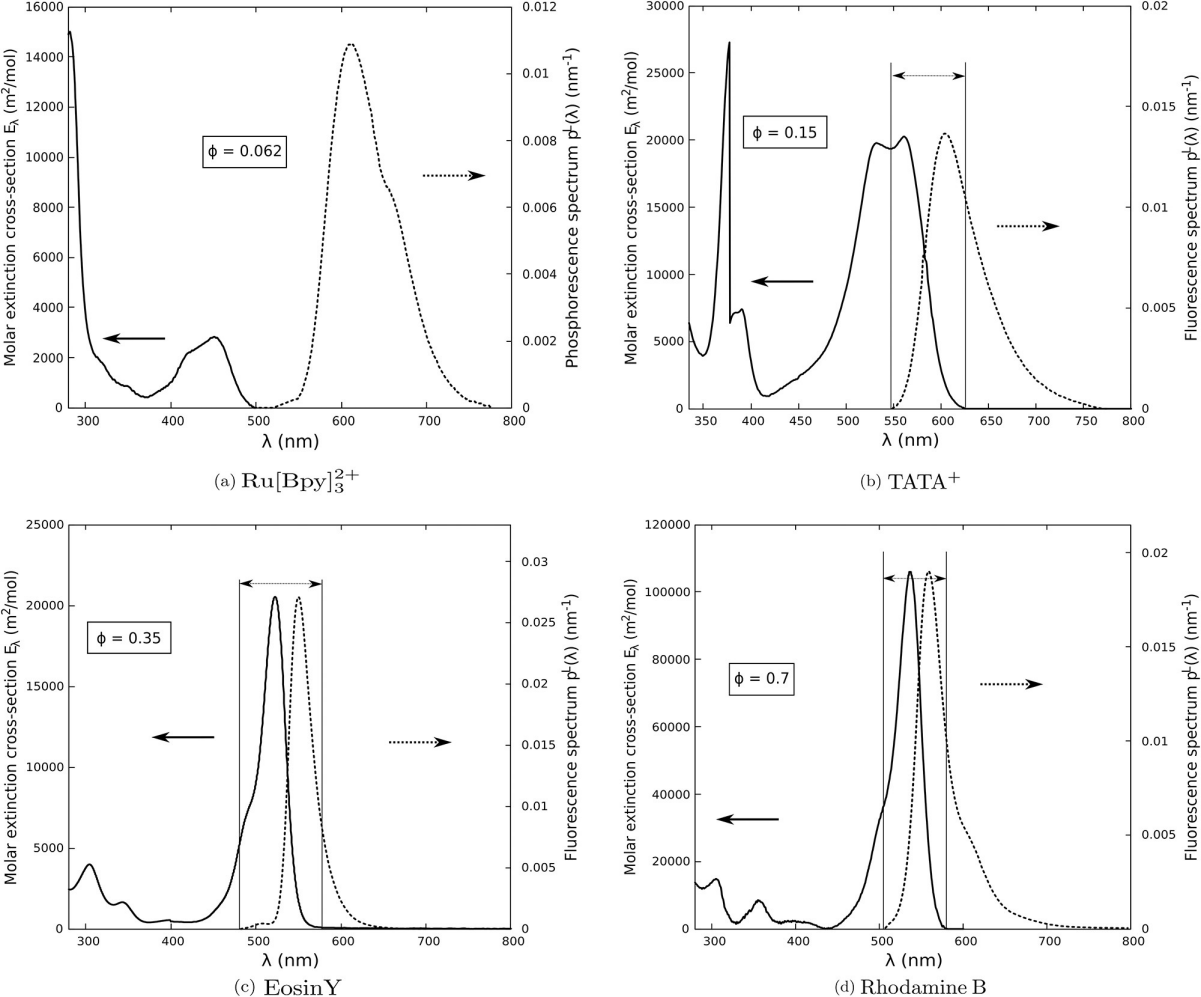
Photosensitizer radiative properties. In all plots, plain lines stand for UV-visible molar extinction cross-section spectrum *E*_λ_ and dashed lines for luminescence emission spectrum *p*^*L*^(λ). Luminescent quantum yield Φ is provided in a box. **(a)** Ru[Bpy]_3_Cl_2_ (40 *μ*M) in aqueous buffer (pH 6.8) solution [48]. **(b)** TATA(Cl) (0.5 mM) UV-visible molar extinction cross-section, TATA(Cl) (10 *μ*M) fluorescence emission spectrum and quantum yield under argon in acetate buffer (1 M) at pH 4.5 [25] **(c)** Eosin Y (24 *μ*M) in a mixture of triethylamine (10% vol.) in water at pH 10.5 [18, 21] **(d)** Rhodamine B in ethanol [26, 49]. Extinction cross-sections are obtained assuming that absorbance *A*_λ_ measured by the authors cited above using spectrophotometry experiments can be interpreted as *A*_λ_ = 1 − exp(−*E*_λ_ *CL*). Overlapping between molar extinction cross-section and luminescence emission spectrum is represented for each photosensitizer (except Ru[Bpy]_3_Cl_2_ for which no overlapping is observed) by 2 parallel lines linked by a double arrow.

**Fig 3:**
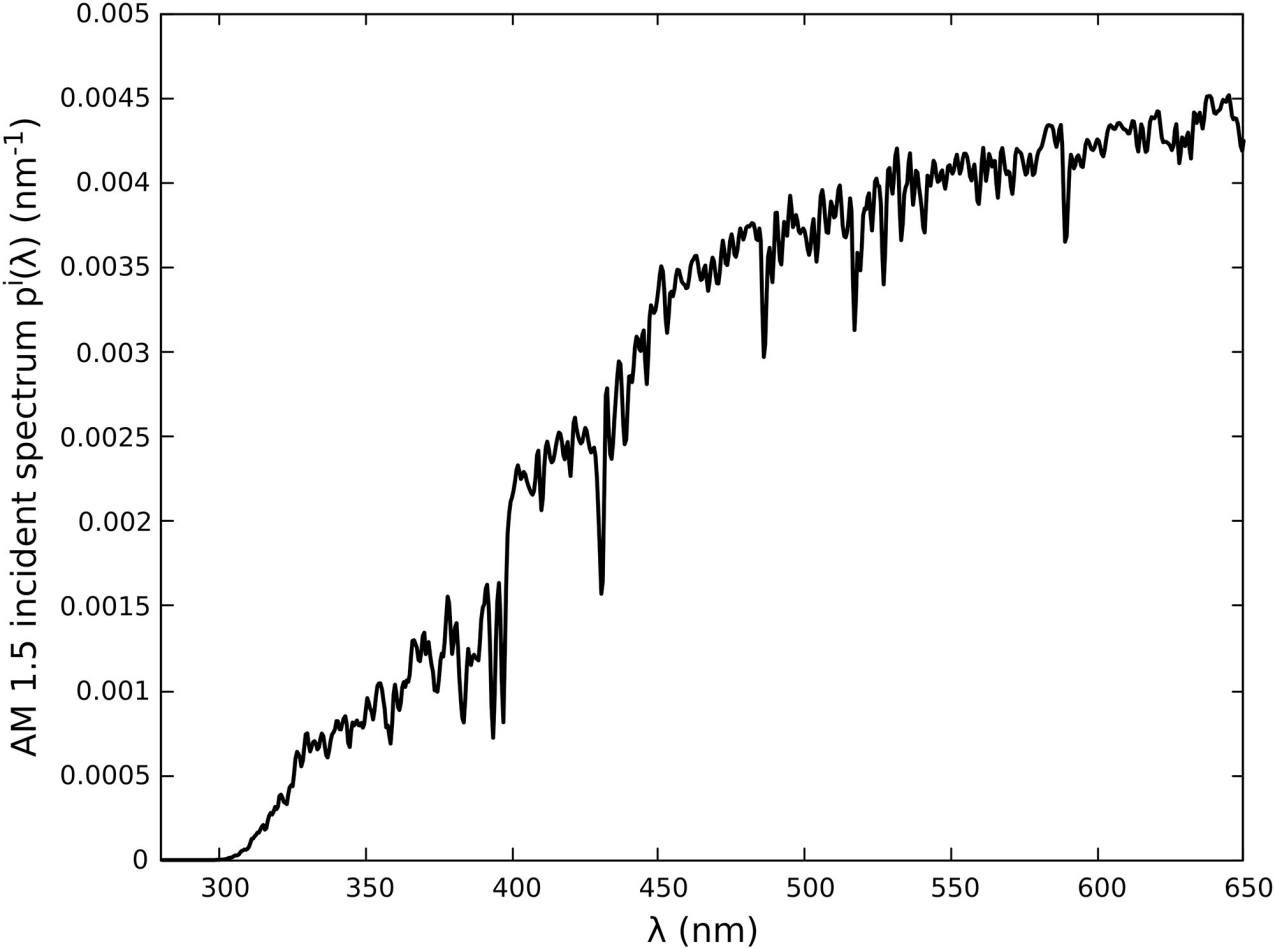
Solar AM1.5 incident spectrum in moles of photons. Probability density function *p*^*i*^(λ) between $\lambda_{min}^{i}=280$ nm and $\lambda_{max}^{i}=65$ nm corresponding to an incident photon flux *q*_0_ = 1743 *μ*mol_*hν*_.m^−2^.s^−1^ (for TATA^+^, we work between $\lambda_{min}^{i}=335$ nm and $\lambda_{max}^{i}=650$ nm and therefore *q*_0_ = 1721 *μ*mol_*hν*_.m^−2^.s^−1^). Note that *p*^*i*^(λ) = 0 for $\lambda \notin [\lambda_{min}^{i},\lambda_{max}^{i}]$ and spectral integration over incident radiation therefore gives $\int_{0}^{+\infty }d\lambda \;p^{i}\left(\lambda \right)•=\int_{\lambda_{min}^{i}}^{\lambda_{max}^{i}}d\lambda \;p^{i}\left(\lambda \right)•$.

Radiative analysis is performed by computing absorptance for incident solar radiation and four commonly-used photosensitizers for solar fuel production: $\text{Ru}{\left[\text{Bpy}\right]}_{3}^{2+}$, TATA^+^, Eosin Y and Rhodamine B. Radiative properties (molar extinction cross-section *E*_λ_, luminescence emission spectra *p*^*L*^(λ) and quantum yield Φ) obtained from the literature are presented in Fig 2. Since these properties depend on the solvent, working conditions were selected to cover a wide range of situations. Incident solar AM1.5 spectrum *p*^*i*^(λ) is used, in the spectral range [280, 650 nm] where the studied photosensitizers are active (see Fig 3). It represents 24.4% of the complete AM1.5 spectrum (in moles of photon distribution, see Fig 3). TATA^+^ is studied in the spectral range [335, 650 nm] only, because molar extinction cross-section is not available in the literature for [280, 335 nm], to the best of our knowledge. In this particular situation it represents a proportion of 24.1% of the total AM1.5 spectrum. Corresponding mean molar extinction cross-sections are provided in Table 1; they are required to evaluate absorption and scattering optical thicknesses with Eqs 4–7. Four incident absorption optical thicknesses were selected: $\tau_{A}^{i}=0.1;0.3;1.4;4.6$. $\tau_{A}^{i}=4.6$ is a photochemistry standard corresponding to 99% of incident photons absorbed in gray and purely absorbing media [1] and other values are chosen to investigate thinner optical thicknesses. In practice, the product *CL* leading to targeted $\tau_{A}^{i}$ values is determined with Eq 4 where ${\bar{E}}^{i}$, ${\bar{E}}^{L}$ and Φ are known for each photosensitizer. Then the same *CL* value is used to compute the scattering optical thickness $\tau_{S}^{L}$ with Eq 6.

**Table 1:** The table summarizes mean molar extinction cross-sections for each photosensitizer. $\bar{E^{i}}$ is weighted by the incident AM1.5 source in the spectral range [280 nm, 650 nm] for $\text{Ru}{\left[\text{Bpy}\right]}_{3}^{2+}$, Eosin Y and Rhodamine B, and in the spectral range [335 nm, 650 nm] for TATA^+^ due to extinction coefficient data availability (see Eq 5 and Fig 3). $\bar{E^{L}}$ is weighted by the luminescence emission spectra (see Eq 7). Note that *p*^*L*^(λ) = 0 for $\lambda \notin [\lambda_{min}^{L},\lambda_{max}^{L}]$, with $\lambda_{min}^{L}=500$ nm and $\lambda_{max}^{L}=800$ nm for $\text{Ru}{\left[\text{Bpy}\right]}_{3}^{2+}$, $\lambda_{min}^{L}=550$ nm and $\lambda_{max}^{L}=775$ nm for TATA^+^, $\lambda_{min}^{L}=475$ nm and $\lambda_{max}^{L}=675$ nm for Eosin Y and $\lambda_{min}^{L}=500$ nm and $\lambda_{max}^{L}=800$ nm for Rhodamine B. Thus spectral integration over luminescence spectrum gives $\int_{0}^{+\infty }d\lambda \;p^{L}\left(\lambda \right)•=\int_{\lambda_{min}^{L}}^{\lambda_{max}^{L}}d\lambda \;p^{L}\left(\lambda \right)•$. ${\bar{E}}^{L}=0$ for $\text{Ru}{\left[\text{Bpy}\right]}_{3}^{2+}$ because no overlapping is observed between extinction cross section and luminescence spectra (see Fig 2).

Photosensitizers	$\text{Ru}{\left[\text{Bpy}\right]}_{3}^{2+}$	TATA^+^	Eosin Y	Rhodamine B
${\bar{E}}^{i}$ (m^2^.mol^−1^)	564	7957	3454	18337
${\bar{E}}^{L}$ (m^2^.mol^−1^)	0	2833	2728	24943

Results gathered in Tables 2 and 3 include:

- absorptance $P_{A}$ provided by the reference model in Section 2, solved with the Monte Carlo algorithm presented in Section 2.5,
- MVRPA 〈A〉 obtained from Eq 23, for a slab of thickness *L* = 1 cm and incident photon flux density *q*_0_ = 1743 *μ*mol_*hν*_.m^−2^.s^−1^, which matches with common laboratory configurations (*q*_0_ = 1721 *μ*mol_*hν*_.m^−2^.s^−1^ will be used for TATA^+^, due to its slightly narrower spectral range). 〈A〉 for any other configuration can easily be obtained by the reader from Eq 23,
- relative difference
  $$\begin{array}{c} \Delta \left(X\right)=\frac{P_{A}^{X}-P_{A}}{P_{A}} \end{array}$$ (29)
  between reference results $P_{A}$ and $P_{A}^{X}$ values obtained with a model denoted *X*. Note that the relative difference for 〈A〉 is equal to that for $P_{A}$ (see Eq 23),
- probability *P*^(*j*)^ that an absorbed photon is absorbed after *j* scattering events (in Table 3), calculated according to Eq 26 where $P_{A}$ and $P_{A}^{\left(j\right)}$ are reference solutions obtained with the Monte Carlo algorithm presented in Section 2.5.

**Table 2:** Results for $\text{Ru}{\left[\text{Bpy}\right]}_{3}^{2+}$, TATA^+^, Eosin Y and Rhodamine B photosensitizers (radiative properties are in Fig 2) for the incident solar spectrum in Fig 3, at selected optical thicknesses $\tau_{A}^{i}$ and $\tau_{S}^{L}$ (see Eqs 4–7 and Table 1): Reference absorptance $P_{A}$, reference MVRPA 〈A〉 (slab thickness *L* = 1 *cm*; incident flux density *q*_0_ = 1743 *μ*mol_*hν*_.m^−2^.s^−1^ and 1721 *μ*mol_*hν*_.m^−2^.s^−1^ for TATA^+^), relative difference Δ(*X*) between these values and those obtained with a model denoted *X* (see Eq 29). *X* ≡ (Φ = 0) when luminescence is neglected, *X* ≡ (*j* = 0) for zero-order scattering expansion ($P_{A}^{X}=P_{A}^{\left(0\right)}$) as presented in Section 4.1, *X* ≡ (*j* ≤ 1) for first-order scattering expansion ($P_{A}^{X}=P_{A}^{\left(0\right)}+P_{A}^{\left(1\right)}$) as presented in Section 4.3, *X* ≡ (*elastic*) and *X* ≡ (*gray*) for elastic and gray models presented in Section 3.3, $X\equiv (\hat{\text{P1}})$ and $X\equiv (\bar{\text{P1}})$ for elastic and gray P1 approximations presented in Section 4.2 and finally, $X\equiv (\bar{\text{SS}})$ for the gray single-scattering approximation presented in Section 4.3. Results for $\text{Ru}{\left[\text{Bpy}\right]}_{3}^{2+}$ do not include all *X* models, since zero-order expansion already provides a reference solution. The relative uncertainty of the Monte Carlo absorptance estimation is always lower than 1%; the precision indicated accounts for case-by-case absolute uncertainty.

**Photosensitizer**	$\text{Ru}{\left[\text{Bpy}\right]}_{3}^{2+}$ (Φ = 0.062)			
$\tau_{A}^{i}\;|\;\tau_{S}^{L}$	0.1 | 0	0.3 | 0	1.4 | 0	4.6 | 0
$P_{A}$	0.0831	0.1805	0.3033	0.3362
〈A〉 [mol_*hν*_.m^−3^.s^−1^]	1.45 × 10^−2^	3.15 × 10^−2^	5.28 × 10^−2^	5.86 × 10^−2^
Δ(Φ = 0)	6.6%	6.6%	6.6%	6.6%
Δ(*j* = 0)	0%	0%	0%	0%
**Photosensitizer**	**TATA**^+^ (Φ = 0.15)			
$\tau_{A}^{i}$ | $\tau_{S}^{L}$	0.1 | 0.006	0.3 | 0.019	1.4 | 0.088	4.6 | 0.289
$P_{A}$	0.0909	0.2265	0.5164	0.6832
〈A〉 [mol_*hν*_.m^−3^.s^−1^]	1.56 × 10^−2^	3.89 × 10^−2^	8.89 × 10^−2^	1.17 × 10^−1^
Δ(Φ = 0)	16.6%	15.4%	13.1%	11.4%
Δ(*j* = 0)	-0.9%	-1.7%	-3.8%	-5.2%
Δ(*j* ≤ 1)	0%	0%	0%	-0.2%
Δ(elastic)	0.2%	0.5%	0.4%	0.4%
$\Delta (\hat{\text{P1}})$	0.5%	0.5%	0.2%	0%
Δ(gray)	0.3%	1.1%	3.4%	5.8%
$\Delta (\bar{\text{P1}})$	-0.5%	-0.3%	2.3%	5.5%
$\Delta (\bar{\text{SS}})$	0.2%	0.9%	2.9%	4.6%
**Photosensitizer**	**Eosin Y** (Φ = 0.35)			
$\tau_{A}^{i}$ | $\tau_{S}^{L}$	0.1 | 0.043	0.3 | 0.128	1.4 | 0.595	4.6 | 1.956
$P_{A}$	0.0818	0.1751	0.3397	0.4934
〈A〉 [mol_*hν*_.m^−3^.s^−1^]	1.43 × 10^−2^	3.05 × 10^−2^	5.92 × 10^−2^	8.59 × 10^−1^
Δ(Φ = 0)	46.5%	40.2%	29.5%	21.5%
Δ(*j* = 0)	-5.3%	-9.3%	-15.9%	-21.1%
Δ(*j* ≤ 1)	-0.6%	-1.1%	-2.6%	-4.7%
Δ(elastic)	0.5%	1.1%	1.7%	1.5%
$\Delta (\hat{\text{P1}})$	-1.5%	-1.2%	-0.2%	1.1%
Δ(gray)	1.7%	4.7%	11.4%	11.4%
$\Delta (\bar{\text{P1}})$	-1.2%	0.8%	9.9%	10.7%
$\Delta (\bar{\text{SS}})$	0.7%	2.0%	2.5%	-3.0%
**Photosensitizer**	**Rhodamine B** (Φ = 0.7)			
$\tau_{A}^{i}$ | $\tau_{S}^{L}$	0.1 | 0.317	0.3 | 0.952	1.4 | 4.444	4.6 | 14.600
$P_{A}$	0.0752	0.1379	0.2288	0.2935
〈A〉 [mol_*hν*_.m^−3^.s^−1^]	1.31 × 10^−2^	2.40 × 10^−2^	3.99 × 10^−2^	5.12 × 10^−2^
Δ(Φ = 0)	170%	142%	116.5%	105.5%
Δ(*j* = 0)	-19.1%	-27.4%	-35.0%	-38.3%
Δ(*j* ≤ 1)	-3.7%	-7.8%	-12.7%	-15.2%
Δ(elastic)	7.7%	16.8%	24.2%	26.4%
$\Delta (\hat{\text{P1}})$	3.3%	13.4%	22.2%	26.3%
Δ(gray)	14.6%	37.7%	68.2%	70.3%
$\Delta (\bar{\text{P1}})$	5.5%	30.6%	65.9%	69.6%
$\Delta (\bar{\text{SS}})$	4.6%	6.2%	2.6%	-1.1%

**Table 3:** Successive orders of scattering for $\text{Ru}{\left[\text{Bpy}\right]}_{3}^{2+}$, TATA^+^, Eosin Y and Rhodamine B photosynthetizers (radiative properties are presented in Fig 2) at selected optical thicknesses $\tau_{A}^{i}$ and $\tau_{S}^{L}$ (see Eqs 4–7 and Fig 2): distribution $P^{\left(j\right)}=P_{A}^{\left(j\right)}/P_{A}$ of scattering orders (or the probability that an absorbed photon is absorbed after *j* scattering events) and cumulative tail distribution $P^{\left(j>q\right)}=\sum_{j=q+1}^{+\infty }P^{\left(j\right)}$ are indicated with a gray background (see Eq 26). *P*^(*j*>*q*)^ is the relative error on both absorptance $P_{A}$ and MVRPA 〈A〉 when the expansion in successive scattering orders is truncated at the *q*-th order. The weight of luminescence radiation is *P*^(*j*>0)^ = *P*^(*S*)^.

**Photosensitizer**	$\text{Ru}{\left[\text{Bpy}\right]}_{3}^{2+}$ (Φ = 0.062)			
$\tau_{A}^{i}\;|\;\tau_{S}^{L}$	0.1 | 0	0.3 | 0	1.4 | 0	4.6 | 0
*P*^(0)^	1	1	1	1
*P*^(*j*>0)^ = *P*^(*S*)^	0	0	0	0
**Photosensitizer**	**TATA**^+^ (Φ = 0.15)			
$\tau_{A}^{i}$ | $\tau_{S}^{L}$	0.1 | 0.006	0.3 | 0.019	1.4 | 0.088	4.6 | 0.289
*P*^(0)^	0.991	0.983	0.962	0.948
*P*^(1)^	0.009	0.019	0.037	0.049
*P*^(*j*>0)^ = *P*^(*S*)^	0.009	0.017	0.038	0.052
*P*^(*j*>1)^	0	0	0	0.002
**Photosensitizer**	**Eosin Y** (Φ = 0.35)			
$\tau_{A}^{i}$ | $\tau_{S}^{L}$	0.1 | 0.043	0.3 | 0.128	1.4 | 0.595	4.6 | 1.956
*P*^(0)^	0.947	0.907	0.841	0.789
*P*^(1)^	0.048	0.082	0.133	0.164
*P*^(2)^	0.002	0.007	0.022	0.036
*P*^(*j*>0)^ = *P*^(*S*)^	0.053	0.093	0.159	0.211
*P*^(*j*>1)^	0.006	0.011	0.026	0.047
*P*^(*j*>2)^	0.003	0.004	0.004	0.011
**Photosensitizer**	**Rhodamine B** (Φ = 0.7)			
$\tau_{A}^{i}$ | $\tau_{S}^{L}$	0.1 | 0.317	0.3 | 0.952	1.4 | 4.444	4.6 | 14.600
*P*^(0)^	0.809	0.726	0.650	0.617
*P*^(1)^	0.154	0.196	0.223	0.232
*P*^(2)^	0.030	0.054	0.080	0.091
*P*^(3)^	0.006	0.016	0.029	0.036
*P*^(*j*>0)^ = *P*^(*S*)^	0.191	0.274	0.350	0.383
*P*^(*j*>1)^	0.037	0.078	0.127	0.152
*P*^(*j*>2)^	0.007	0.024	0.047	0.061
*P*^(*j*>3)^	0	0.008	0.018	0.025

The conclusions of this analysis provide guidelines to construct relevant radiative transfer approximations in Section 4.

### 3.1 Overall luminescence effect

Here we compare absorptance values $P_{A}^{\left(\Phi =0\right)}$ obtained when luminescence is neglected, *i.e*. when Φ is set to zero in our model, with $P_{A}$ values obtained from the reference model. The results in Table 2 present the relative difference *Δ*(Φ = 0) computed with Eq 29, where *X* ≡ (Φ = 0).

Neglecting luminescence always leads to an overestimation of absorptance: Δ(Φ = 0)>0. Indeed when luminescence is not taken into account, each interaction with photosensitizers necessarily leads to absorption, whereas luminescence interaction has a probability Φ of leading to scattered photons that might exit the medium. We record Δ(Φ = 0) values between 10% and 20% for TATA^+^, between 20% and 50% for Eosin Y and larger than 100% for Rhodamine B. The only photosensitizer for which Δ(Φ = 0) is constant and lower than 10% is $\text{Ru}{\left[\text{Bpy}\right]}_{3}^{2+}$, but we will see in the next paragraphs and in Section 4 that strictly accounting for luminescence is very simple in this particular case. As the absorption optical thickness $\tau_{A}^{i}$ increases, absorptance given by both models increases (see $P_{A}$ values in Table 2) and the error Δ(Φ = 0) decreases. Nevertheless, Δ(Φ = 0) does not approach 0 when photosensitizer concentration goes to infinity. Indeed, reference $P_{A}$ approaches a limit value lower than that for $P_{A}^{\left(\Phi =0\right)}$, because luminescence radiation cannot be completely absorbed, for two reasons. The first reason is caused by inelastic scattering: part of the luminescence radiation belongs to a spectral range where the extinction cross section is equal to zero (see Fig 2 and Section 2.3) and therefore photons exit the medium without interacting with photosensitizer. The second is driven by multiple scattering: some photons are backscattered in the vicinity of *x* = 0 and exit the medium through the front face of the reactor (even when concentration goes to infinity, or equivalently, for a semi-infinite medium).

Overall, these results indicate significant luminescence effects that should not be neglected when studying photosensitized systems.

#### 3.1.1 Remark on spectral effects that are not due to luminescence

In Table 2, for absorption optical thickness $\tau_{A}^{i}=4.6$ it could be expected that $P_{A}^{\left(\Phi =0\right)}=0.99$ [1] but this is not the case: $P_{A}^{\left(\Phi =0\right)}=0.358$ for $\text{Ru}{\left[\text{Bpy}\right]}_{3}^{2+}$, $P_{A}^{\left(\Phi =0\right)}=0.761$ for TATA^+^, $P_{A}^{\left(\Phi =0\right)}=0.599$ for Eosin Y and $P_{A}^{\left(\Phi =0\right)}=0.603$ for Rhodamine B. Indeed, $\tau_{A}^{i}=4.6$ leads to 99% of incident photons being absorbed only for gray media (or monochromatic incident radiation). Here we choose to compute absorptance in the spectral range [280 nm, 650 nm] (or [335 nm, 650nm] for TATA^+^), but the molar extinction coefficient is equal to zero over part of this spectral range (see Fig 2). Therefore, even when $\tau_{A}^{i}$ approaches infinity, absorptance is significantly lower than 0.99. Nevertheless, when $\tau_{A}^{i}$ = 4.6 most of the incident radiation belonging to a spectral range for which *E*_λ_ ≠ 0 interacts with the medium. If for each photosensitizer absorptance $P_{A}^{\left(\Phi =0\right)}$ is calculated in the spectral range where *E*_λ_ ≠ 0, we obtain: 0.96 for $\text{Ru}{\left[\text{Bpy}\right]}_{3}^{2+}$, 0.86 for TATA^+^, 0.82 for Eosin Y and 0.86 for Rhodamine B. The difference with 0.99 is due to second-order spectral effects that are not related to luminescence. Therefore, those effects will not be discussed further in this article that focuses on luminescence.

### 3.2 Successive orders of scattering analysis

Here we analyze the effect of multiple luminescence scattering. In the previous paragraphs we discussed the fact that due to luminescence, interaction has a probability Φ of leading to scattered photons that might exit the medium. Therefore $P_{A}$ is decreased compared to situations without luminescence, leading to a loss with respect to the photoreaction. However, those scattered photons emitted by luminescence do not necessarily exit the medium. They can interact (again) with photosensitizers and be absorbed—in this case they participate in the reaction—or scattered, and so on.

Table 3 presents the distribution *P*^(*j*)^ of scattering orders and its tail cumulative *P*^(*j*>*q*)^ (see Eq 26): *P*^(0)^ is the contribution of ballistic photons, *P*^(1)^ is the contribution of photons absorbed after one scattering event (first order), *P*^(2)^ is the contribution of photons absorbed after two scattering events (second order), *etc*., with decreasing contributions *P*^(*j*)^ > *P*^(*j*+1)^ and normalization $\sum_{j=0}^{+\infty }P^{\left(j\right)}=1$. The key parameter in analyzing this distribution is the scattering optical thickness $\tau_{S}^{L}$. The absorption of luminescence radiation increases with optical thickness, leading to a higher luminescence contribution *P*^(*j*>0)^ = *P*^(*S*)^ and lower ballistic contribution *P*^(0)^ (the distribution is normalized). Furthermore, as regards luminescence contributions, the higher the $\tau_{S}^{L}$, the larger the contribution of high scattering orders.

The following case-by-case analysis is organized according to $\tau_{S}^{L}$ value, which is strongly influenced by the overlap between extinction cross-section and luminescence emission spectra *via* $\bar{E^{L}}$ (see Eqs 6 and 7 and Fig 2).

#### 3.2.1 Case 1: $\tau_{\text{S}}^{\text{L}}=0$ because extinction and luminescence spectra do not overlap

This case is illustrated here with $\text{Ru}{\left[\text{Bpy}\right]}_{3}^{2+}$ photosensitizer (see Fig 2). The photosensitizer does not interact with scattered photons and there is no multiple scattering. Luminescence is therefore a pure loss for the reaction, since only incident solar radiation participates in absorption. In terms of successive orders of scattering, it translates into *P*^(0)^ = 1 and *P*^(*j*>0)^ = 0 in Table 3, or equivalently, $P_{A}^{\left(S\right)}=0$ and therefore $P_{A}=P_{A}^{\left(0\right)}$ in Eq 25: zero-order expansion is sufficient to rigorously describe luminescence. Indeed, Δ(*j* = 0) = 0 for $\text{Ru}{\left[\text{Bpy}\right]}_{3}^{2+}$. Thus strictly accounting for luminescence is very simple in this particular case, as it will be discussed in Section 4.1.

#### 3.2.2 Case 2: $\tau_{\text{S}}^{\text{L}}\neq 0$ because extinction and luminescence spectra overlap

This is the case for most fluorescent molecules, as illustrated here with TATA^+^, Eosin Y and Rhodamine B. In this situation the photosensitizer interacts with scattered luminescence radiation that can be absorbed after one, two, … *j* scattering events:

- Looking at the TATA^+^ case, ballistic photons *P*^(0)^ are responsible for at least 95% of absorption. The proportion of photons *P*^(*j*>1)^ absorbed after more than one scattering event is lower than 0.002 in the studied cases, indicating that ballistic photons and photons absorbed after one scattering event *P*^(1)^ represent about 99% of photon absorption.
- In the case of Eosin Y, the photon proportion absorbed after more than one scattering event *P*^(*j*>1)^ is lower than 0.05 in the studied cases, indicating that ballistic photons and photons absorbed after one scattering event contribute to 95% of absorption. For $\tau_{S}^{L}=0.043$, we are in the same situation as for TATA^+^: ballistic photons alone are responsible for 95% of absorption, and adding the first scattering order *P*^(1)^ leads to 99%. For the higher optical thicknesses, ballistic photons represent 80 to 90% of absorption. For $\tau_{S}^{L}=0.128$, again, ballistic and first-order *P*^(0)^ + *P*^(1)^ are responsible for 99% of absorption. For $\tau_{S}^{L}=0.595$ and 1.956, ballistic and first-order contribute to more than 95% of absorption. For these two last optical thicknesses, accounting for photons absorbed after 0, 1 and 2 scattering events (second-order expansion) is required to describe 99% of absorption.
- Regarding Rhodamine B, scattering optical thicknesses are significantly higher than for other photosensitizers, due to higher values for luminescence quantum yield and overlapping. Consequently, the contribution of photons absorbed after more than one scattering event *P*^(*j*>1)^ becomes significant. For $\tau_{S}^{L}=0.317$ and 0.952, first-order expansion is sufficient to describe 95% of absorption but the second order is required to describe 99% of absorption. For $\tau_{S}^{L}=4.444$ and 14.6, second-order expansion describes 95% of absorption but the third and fourth orders (photons absorbed after three and four scattering events) are required to describe 99% of absorption.

In summary, these results indicate that in the case investigated here 95% of photons absorbed are:

- ballistic photons only when $\tau_{S}^{L}<0.05$,
- ballistic photons and photons absorbed after one scattering event when $0.05<\tau_{S}^{L}<1$,
- ballistic photons and photons absorbed after one and two scattering events when $\tau_{S}^{L}>1$,

### 3.3 Inelastic scattering sensitivity

Inelastic scattering is another conceptual and numerical difficulty when describing photon transport in photosensitized and photocatalytic systems, in addition to multiple scattering. Indeed, wavelengths are redistributed according to the luminescence spectrum at each scattering event. Here we analyze the sensitivity of absorptance and MVRPA to inelastic scattering effects in order to determine if this difficulty must be tackled or can be partially bypassed. For this purpose, we constructed two equivalent models for luminescence radiation that we compared with the reference solution (see Δ(*gray*) and Δ(*elastic*) in Table 2). In the first one, gray approximation is used in the luminescence spectral range and in the second, the inelastic collision term in the RTE for luminescence intensity is simply replaced by an elastic collision term. In both cases, the spectral distribution for ballistic photons (zero-th order) is preserved; only luminescence intensity is affected by the approximations.

**A gray model for luminescence radiation** is constructed based on the distinction between ballistic and scattered intensity (see Eq 18). Gray approximation is used in the RTE for scattered (*i.e*. luminescence) intensity $I_{\lambda }^{\left(S\right)}\left(x,\mu \right)$, which turns the inelastic collision term into an elastic collision term.

The RTE in Eq 19 is integrated over wavelengths λ and then the following gray approximation is used:

$$\begin{array}{c} \int_{0}^{+\infty }d\lambda \;k_{\lambda }\;I_{\lambda }^{\left(S\right)}\left(x,\mu ;k_{\lambda }\right)≃\bar{k}\;I^{\left(S\right)}\left(x,\mu ;\bar{k}\right) \end{array}$$ (30)

where $I^{\left(S\right)}=\int_{0}^{+\infty }d\lambda \;I_{\lambda }^{\left(S\right)}$ and $\bar{k}=\int_{0}^{+\infty }d\lambda \;p^{L}\left(\lambda \right)\;k_{\lambda }={\bar{E}}^{L}C$ (see Eq 7). Using the normalization $\int_{0}^{+\infty }d\lambda \;p^{L}\left(\lambda \right)=1$ and the notation ${\bar{I}}^{\left(S\right)}$ for this gray approximation leads to

$$\begin{array}{c} \mu \frac{d{\bar{I}}^{\left(S\right)}\left(x,\mu \right)}{dx}=-\bar{k}\;{\bar{I}}^{\left(S\right)}\left(x,\mu \right)+\frac{\Phi \bar{k}}{2}\int_{-1}^{1}d\mu^{\prime }\;{\bar{I}}^{\left(S\right)}\left(x,\mu^{\prime }\right)+\int_{0}^{+\infty }d\lambda^{\prime }\;\frac{\Phi k_{\lambda^{\prime }}}{2}\int_{-1}^{1}d\mu^{\prime }\;I_{\lambda^{\prime }}^{\left(0\right)}\left(x,\mu^{\prime }\right) \end{array}$$ (31)

with the boundary condition

$$\begin{array}{c} {\bar{I}}^{\left(S\right)}\left(0,\mu \right)=0 \end{array}$$ (32)

Note that $I_{\lambda }^{\left(0\right)}$ in Eq 31 is the solution of $\text{Ru}{\left[\text{Bpy}\right]}_{3}^{2+}$ Eq 12: ballistic intensity is not affected by the present approximation. Then the contribution of scattered photons to absorptance is obtained by applying the gray approximation Eq 30 to the definition in Eq 24:

$$\begin{array}{c} {\bar{P}}_{A}^{\left(S\right)}=\frac{1}{q_{0}}\int_{0}^{L}dx\int_{-1}^{1}d\mu \left(1-\Phi \right)\bar{k}\;{\bar{I}}^{\left(S\right)}\left(x,\mu \right) \end{array}$$ (33)

Finally, our gray model for absorptance is obtained by summing the contributions of ballistic and scattered photons:

$$\begin{array}{c} P_{A}^{\;(\text{gray})}=P_{A}^{\left(0\right)}+{\bar{P}}_{A}^{\left(S\right)} \end{array}$$ (34)

where $P_{A}^{\left(0\right)}$ is not modified by the approximation (see its definition in Eq 24).

The $P_{A}^{\;(\text{gray})}$ values presented in Table 2 were obtained with the Monte Carlo algorithm presented in Section 2.5, except that **Step 4a.3** was replaced by:

- **Step 4a.3**: $k_{\lambda }=\bar{k}$

Whatever the wavelength, the extinction coefficient for scattered photons is set to $\bar{k}={\bar{E}}^{L}C$ (see Fig 2).

The relative difference Δ(*gray*) between $P_{A}^{\;(\text{gray})}$ and the reference $P_{A}$ is presented in Table 2. Results do not include $\text{Ru}{\left[\text{Bpy}\right]}_{3}^{2+}$, since luminescence radiation does not participate in absorption in this case (see Section 2.3). For other photosensitizers, the fact that Δ(*gray*) > 0 (always) indicates that gray approximation for luminescence radiation overestimates absorptance (and indeed, $f\left(k_{\lambda }\right)=\int_{0}^{L}dx\int_{-1}^{1}d\mu \;\left(1-\Phi \right)k_{\lambda }I_{\lambda }^{\left(S\right)}\left(x,\mu \right)$ in Eq 24 is concave).

Δ(gray) rapidly increases with the scattering optical thickness $\tau_{S}^{L}$, because the average number of wavelength redistributions due to inelastic scattering events increases with $\tau_{S}^{L}$. Indeed, the analysis in Section 2.3 insists on the significant contribution of scattering orders *j* = 1, 2, 3 in some tested cases, involving *j* wavelength redistribution events (see Table 3). For TATA^+^, Δ (gray) ≲ 5 since at least 95% of absorption is due to ballistic radiation that is not affected by inelastic scattering. For Eosin Y, the first and second scattering orders contribute up to 20% and Δ(*gray*) reaches 11%. For Rhodamine B, we record a significant contribution up to the third scattering order, and Δ(*gray*) reaches 70%.

Δ(gray) always increases with $\tau_{S}^{L}$ for a given set of radiative properties, but the magnitude of that increase depends on the extinction cross section and luminescence spectra. For example, if the extinction cross section is gray within the luminescence spectral range (*i.e*.*E*_λ_ does not vary with λ in the spectral range $\left[\lambda_{min}^{L},\lambda_{max}^{L}\right]$ where *p*^*L*^(λ) ≠ 0), then the gray model and reference model are exactly identical, and therefore $\Delta \left(\text{gray}\right)=0\;\;\forall \;\tau_{S}^{L}$. In general, we expect that the higher the spectral variation of *E*_λ_ on $\left[\lambda_{min}^{L},\lambda_{max}^{L}\right]$ is, the faster Δ(*gray*) increases with $\tau_{S}^{L}$. We cannot illustrate this behavior here because the typical photosensitizers we selected have comparable spectral variations over the luminescence spectral range (see Fig 2).

To conclude, for the typical cases tested in this paper, wavelength redistribution due to luminescence can hardly be put aside once scattering optical thickness exceeds 0.1. However, the results in Table 3 indicate that the main contribution to luminescence radiation is due to first-order terms in scattering expansion. Therefore we hereafter study a model that preserves luminescence spectral redistribution during the first scattering event only, and neglects the subsequent ones.

**The elastic-scattering model for luminescence radiation** is simply obtained by replacing the inelastic collision term in the RTE for luminescence intensity (see Eq 19) by an elastic collision term:

$$\begin{array}{ccc} \mu \frac{d{\hat{I}}_{\lambda }^{\;(S)}\left(x,\mu \right)}{dx} & = & -k_{\lambda }{\hat{I}}_{\lambda }^{\;(S)}\left(x,\mu \right)+\frac{\Phi k_{\lambda }}{2}\int_{-1}^{1}d\mu^{\prime }\;{\hat{I}}_{\lambda }^{\;(S)}\left(x,\mu^{\prime }\right)\\ & + & p^{L}\left(\lambda \right)\int_{0}^{+\infty }d\lambda^{\prime }\frac{\Phi k_{\lambda^{\prime }}}{2}\int_{-1}^{1}d\mu^{\prime }\;I_{\lambda^{\prime }}^{\left(0\right)}\left(x,\mu^{\prime }\right) \end{array}$$ (35)

Luminescence absorptance ${\hat{P}}_{A}^{\left(S\right)}$ is defined in Eq 24 with $I_{\lambda }^{\left(•\right)}={\hat{I}}^{\;(S)}$ and

$$\begin{array}{c} P_{A}^{\;(\text{elastic})}=P_{A}^{\left(0\right)}+{\hat{P}}_{A}^{\left(S\right)} \end{array}$$ (36)

where $P_{A}^{\left(0\right)}$ is not affected by the approximation.

The $P_{A}^{\;(\text{elastic})}$ values presented in Table 2 were obtained using the Monte Carlo algorithm presented in Section 2.5, except that **Step 4a.3** was replaced by:

- **Step 4a.3**:
  **if** *j* = = 1 *then*
  The wavelength λ is sampled according to the luminescence spectrum *p*^*L*^(λ) and the extinction coefficient *k*_λ_ = *CE*_λ_ is interpolated.
  **end**

The wavelength λ is sampled during the first scattering event only (*i.e*. for *j* = 1).

Two spectral integrations are required to solve this model—one over the incident spectrum and one over the luminescence spectrum—whereas only spectral integration over the incident spectrum was considered in the previous gray model. This additional spectral dimension is required to take into account the luminescence spectrum when ballistic photons are scattered and move from population (0) to population (1). The comparison of Δ(*elastic*) and Δ(*gray*) in Table 2 indicates that it is a significant improvement in the description of absorption, and yet it corresponds to a considerable conceptual and practical simplification compared to the reference model, which involves an infinite number of spectral integrations (one at each scattering order). This simplified description of inelastic scattering provides results with a relative difference Δ(*elastic*) below 2% for TATA^+^ and Eosin Y. This is due to the fact that scattering orders *j* > 1 have little impact with these photosensitizers (see Table 3). For Rhodamine, however, these scattering orders contribute up to 15% of absorptance, in addition to the strong spectral variation in the extinction cross section in the luminescence spectral range $\left[\lambda_{min}^{L},\lambda_{max}^{L}\right]$ (see Fig 2d). In this case, inelastic scattering should be described in all its complexity.

## 4 Analytical approximate solutions

Based on the above radiative analysis, we propose in the present section to develop five approximations and discuss their behavior. We aim at providing meaningful analytical solutions enabling a simple evaluation and analysis of luminescence radiation in most cases, without having to use advanced numerical methods. This work is based on zero-order scattering, P1 and single-scattering approximations. But first let us clarify a common practice in photochemistry, which consists in neglecting luminescence.

**Neglecting luminescence** is equivalent to taking Φ = 0 in the reference RTE Eq 8, with the same boundary conditions as in Eq 9:

$$\begin{array}{c} \mu \frac{dI_{\lambda }^{\left(\Phi =0\right)}\left(x,\mu \right)}{dx}=-k_{\lambda }\;I_{\lambda }^{\left(\Phi =0\right)}\left(x,\mu \right) \end{array}$$ (37)

leading to a simple Bouguer exponential attenuation along straight paths:

$$\begin{array}{ccc} I_{\lambda }^{\left(\Phi =0\right)}\left(x,\mu =1\right) & = & q_{0,\lambda }\;e^{-k_{\lambda }\;x} \end{array}$$ (38)

$$\begin{array}{ccc} I_{\lambda }^{\left(\Phi =0\right)}\left(x,\mu \neq 1\right) & = & 0 \end{array}$$ (39)

Substituting this expression into Eq 24 with Φ = 0 leads to

$$\begin{array}{c} P_{A}^{\left(\Phi =0\right)}=\int_{0}^{+\infty }d\lambda \;p^{i}\left(\lambda \right)\;\left(1-e^{-k_{\lambda }L}\right) \end{array}$$ (40)

With this model, the extinction coefficient *k*_λ_ is equal to the absorption coefficient, and radiation is collimated along the **e**_*x*_ axis (no deviation due to scattering here).

As discussed in Section 3.1, we observe serious Δ(Φ = 0) errors when neglecting luminescence (see Table 2). A straightforward and significant improvement is obtained by using instead the 0^*th*^ order scattering approximation.

### 4.1 Zero-order scattering approximation

In Section 3.2 we observed that the main contribution to absorption is due to ballistic radiation. The expansion in successive orders of scattering is therefore truncated here at order zero: $P_{A}≃P_{A}^{\left(0\right)}$. In comparison with the reference solution in Eq 25, the absorption of luminescence radiation $P_{A}^{\left(S\right)}$ is not taken into account, leading to quite a simple analytic expression: the solution of the RTE Eq 12 for ballistic intensity $I_{\lambda }^{\left(0\right)}$ is

$$\begin{array}{ccc} I_{\lambda }^{\left(0\right)}\left(x,\mu =1\right) & = & q_{0,\lambda }\;e^{-k_{\lambda }x} \end{array}$$ (41)

$$\begin{array}{ccc} I_{\lambda }^{\left(0\right)}\left(x,\mu \neq 1\right) & = & 0 \end{array}$$ (42)

and substituting this expression into Eq 24 leads to

$$\begin{array}{c} P_{A}^{\left(0\right)}=\left(1-\Phi \right)\int_{0}^{+\infty }d\lambda \;p^{i}\left(\lambda \right)\;\left(1-e^{-k_{\lambda }L}\right)=\left(1-\Phi \right)P_{A}^{\left(\Phi =0\right)} \end{array}$$ (43)

Let us emphasize that $P_{A}^{\left(0\right)}$ is very easily obtained by multiplying $P_{A}^{\left(\Phi =0\right)}$, which is routinely calculated or measured in photochemistry practice, by (1 − Φ).

The solution for $I_{\lambda }^{\left(0\right)}$ is the same as for $I_{\lambda }^{\left(\Phi =0\right)}$, but a crucial difference is that the luminescence quantum yield Φ is here taken into account in the expression of $P_{A}$ and 〈A〉. When luminescence is neglected, every photon that interacts with the photosensitizer is counted as being absorbed, leading to an overestimation of $P_{A}$: Δ(Φ = 0) > 0 in Table 2. Here, in contrast, luminescence scattering is modeled but only ballistic photon absorption is accounted for, leading to the prefactor (1 − Φ) in Eq 43. Since the absorption of luminescence radiation is ignored, the zero-order scattering approximation underestimates absorptance: Δ(*j* = 0) < 0 in Table 2. The impact of truncating the expansion in successive orders of scattering has already been discussed in Sections 2.3 and 3.2, and indeed, Δ(*j* = 0) in Table 2 is by definition equal to −*P*(*j* > 0) in Table 3. As seen in previous discussions, the error associated with zero-order scattering approximation is governed by the scattering optical thickness $\tau_{S}^{L}$: the higher $\tau_{S}^{L}$ is, the higher the error Δ(*j* = 0).

Of course, when $\tau_{S}^{L}=0$, the zero-order scattering approximation gives exact results: $I_{\lambda }=I_{\lambda }^{\left(0\right)}$, $\langle A\rangle ={\langle A\rangle }^{\left(0\right)}$ and $P_{A}=P_{A}^{\left(0\right)}$. This situation is encountered in two cases. First, for photosensitizers that do not emit luminescence radiation (*i.e*. when Φ = 0). In this case $I_{\lambda }=I_{\lambda }^{\left(\Phi =0\right)}=I_{\lambda }^{\left(0\right)}$, $\langle A\rangle ={\langle A\rangle }^{\left(\Phi =0\right)}={\langle A\rangle }^{\left(0\right)}$ and $P_{A}=P_{A}^{\left(\Phi =0\right)}=P_{A}^{\left(0\right)}$. Second, and more importantly, for photosensitizers with extinction cross-section and luminescence emission spectra that do not overlap, as illustrated with $\text{Ru}{\left[\text{Bpy}\right]}_{3}^{2+}$ in Section 3.1 (see Case 1). In this case, the error obtained with a model neglecting luminescence is $\Delta \left(\Phi =0\right)=\left(P_{A}^{\left(\Phi =0\right)}-P_{A}^{\left(0\right)}\right)/P_{A}^{\left(0\right)}=\Phi /\left(1-\Phi \right)$. In Table 2, indeed, Δ(Φ = 0) = 6.6% for $\text{Ru}{\left[\text{Bpy}\right]}_{3}^{2+}$ with luminescence quantum yield Φ = 0.062. Note that a photosensitizer with a higher Φ could lead to a significantly greater error: *e.g*. Δ(Φ = 0) = 25% for Φ = 0.2. In these situations where $\tau_{S}^{L}=0$, the very simple solution provided in Eq 43 should be used.

In Table 2, zero-order scattering approximation is also tested for three photosensitizers with $\tau_{S}^{L}\neq 0$: TATA^+^, Eosin Y and Rhodamine B. As discussed in Section 3.1, the luminescence effect is significant in these cases and should not be neglected (Δ(Φ = 0) = 10% to 170%):

- For TATA^+^, the zero-order scattering approximation provides accurate results with error Δ(*j* = 0) ≤ 5% in every tested situation. Indeed, Table 3 indicates that at least 95% of absorption is due to ballistic photons. The relative error is 2 to 20 times lower than the error Δ(Φ = 0) obtained when ignoring luminescence.
- For Eosin Y and Rhodamine B, even if the zero-order scattering approximation is not sufficient to describe photon transport with accuracy below 5%, we nevertheless record a significant improvement in relative error compared to Δ(Φ = 0).

Overall, we record the following accuracy levels for the zero-order scattering approximation as a function of scattering optical thickness $\tau_{S}^{L}$:

- Δ(*j* = 0) < 5% for $\tau_{S}^{L}$ < 0.05,
- Δ(*j* = 0) < 10% for 0.05 $<\tau_{S}^{L}$ < 0.3,
- Δ(*j* = 0) < 30% for 0.3 < $\tau_{S}^{L}$ < 1.

In conclusion, zero-order scattering is a relevant approximation that provides accurate results for optical thicknesses $\tau_{S}^{L}<0.05$ and for TATA^+^ in general; it includes half the cases investigated in this paper. In addition, this approximation is exceedingly simple because it only requires multiplying $P_{A}^{\left(\Phi =0\right)}$, which is routinely calculated or measured in photochemistry practice, by (1 − Φ).

### 4.2 P1 approximations for luminescence radiation

For optical thicknesses $\tau_{S}^{L}>0.05$, the absorption of luminescence radiation must be described to improve accuracy compared to zero-order approximation. Here, the luminescence radiation $P_{A}^{\left(S\right)}$ in Eq 25 is described by diffusion equations derived thanks to P1 approximation [35, 50]. But a simple analytical solution is only accessible for elastic scattering processes, and therefore P1 approximation is applied to the gray and elastic models presented in Section 3.3.

#### 4.2.1 P1 approximation applied to the gray model for luminescence radiation

The gray approximation presented in Section 3.3 produces fairly accurate results for TATA^+^ and Eosin Y (see Δ(*gray*) in Table 2) and is also expected to be relevant in the case of photosensitizers with small spectral variations in extinction cross section over the luminescence spectral range. Therefore the P1 approximation is applied to the gray RTE in Eq 31. The trivial reference solution for ballistic photons (order zero) is retained, including incident spectral distribution, but wavelength redistribution due to luminescence is not taken into account: $P_{A}^{\left(S\right)}≃{\bar{P}}_{A}^{\left(S\right)}$. Hereafter, a simple analytic expression for P1 approximation $P_{A}^{\left(S\right)}≃{\bar{P}}_{A}^{\left(S,P1\right)}$ is obtained and we study:

$$\begin{array}{c} P_{A}≃P_{A}^{\left(\bar{\text{P1}}\right)}=P_{A}^{\left(0\right)}+{\bar{P}}_{A}^{\left(S,P1\right)} \end{array}$$ (44)

where $P_{A}^{\left(0\right)}$ is the reference zero-order absorptance given in Eq 43 and ${\bar{P}}_{A}^{\left(S,P1\right)}$ is the luminescence absorptance resulting from both gray and P1 approximations (see Eq 53).

First the expression of $I_{\lambda }^{\left(0\right)}$ in Eq 41 is substituted into the gray RTE Eq 31:

$$\begin{array}{c} \mu \frac{d{\bar{I}}^{\left(S\right)}}{dx}=-\bar{k}\;{\bar{I}}^{\left(S\right)}\left(x,\mu \right)+\frac{\Phi \bar{k}}{2}\int_{-1}^{1}d\mu^{\prime }\;{\bar{I}}^{\left(S\right)}\left(x,\mu^{\prime }\right)+\int_{0}^{+\infty }d\lambda^{\prime }\;\frac{\Phi k_{\lambda^{\prime }}}{2}q_{0,\lambda^{\prime }}\;e^{-k_{\lambda^{\prime }}x} \end{array}$$ (45)

The first moment of this equation is integrated over *μ* ∈ [−1, 1] to obtain the local macroscopic balance:

$$\begin{array}{c} \frac{d\;{\bar{q}}^{\;(S)}}{dx}=-\bar{k}\left(1-\Phi \right))\;{\bar{G}}^{\;(S)}\left(x\right)+\int_{0}^{+\infty }d\lambda^{\prime }\;\Phi k_{\lambda^{\prime }}q_{0,\lambda^{\prime }}\;e^{-k_{\lambda^{\prime }}x} \end{array}$$ (46)

where ${\bar{G}}^{\;(S)}\left(x\right)=\int_{-1}^{1}d\mu \;{\bar{I}}^{\left(S\right)}\left(x,\mu \right)$ is the irradiance and ${\bar{q}}^{\;(S)}\left(x\right)=\int_{-1}^{1}d\mu \;{\bar{I}}^{\left(S\right)}\left(x,\mu \right)\;\mu$ is the surface flux density at location *x*. Afterwards, Eq 45 is multiplied by *μ* and then integrated over *μ* ∈ [−1, 1]:

$$\begin{array}{c} \int_{-1}^{1}d\mu \;\mu^{2}\frac{d{\bar{I}}^{\left(S\right)}}{dx}=-\bar{k}\;{\bar{q}}^{\;(S)}\left(x\right) \end{array}$$ (47)

P1 approximation assumes linear angular dependence of intensity:

$$\begin{array}{c} {\bar{I}}^{\left(S\right)}\left(x,\mu \right)=a\left(x\right)+b\left(x\right)\;\mu \end{array}$$ (48)

This functional form of intensity is substituted into the left-hand term of Eq 47, and integration leads to Fick’s law of diffusion [35, 50]:

$$\begin{array}{c} {\bar{q}}^{\;(S,P1)}\left(x\right)=-\frac{1}{3\;\bar{k}}\frac{d{\bar{G}}^{\;(S,P1)}}{dx} \end{array}$$ (49)

Finally, substituting Eq 49 in the local balance Eq 46 gives the diffusion equation:

$$\begin{array}{c} \frac{d^{2}\;{\bar{G}}^{\;(S,P1)}}{dx^{2}}-3\;{\bar{k}}^{2}\left(1-\Phi \right)\;{\bar{G}}^{\;(S,P1)}\left(x\right)=-\int_{0}^{+\infty }d\lambda \;3\;\Phi \bar{k}\;k_{\lambda }q_{0,\lambda }\;e^{-k_{\lambda }x} \end{array}$$ (50)

In this work, the Marshak boundary conditions detailed in [50] are used:

$$\begin{array}{ccc} {\bar{G}}^{\;(S,P1)}\left(0\right)-\frac{2}{3\;\bar{k}}\frac{d{\bar{G}}^{\;(S,P1)}}{dx}\left(0\right) & = & 0\\ {\bar{G}}^{\;(S,P1)}\left(L\right)+\frac{2}{3\;\bar{k}}\frac{d{\bar{G}}^{\;(S,P1)}}{dx}\left(L\right) & = & 0 \end{array}$$ (51)

The solution to this equation is derived in S1 Appendix and used in the definition of luminescence absorptance (Eq 33). After symbolic integration, we obtain the expression of total absorptance in Eq 44 where:

$$\begin{array}{ccc} {\bar{P}}_{A}^{\left(S,P1\right)} & = & \int_{0}^{+\infty }d\lambda \;p^{i}\left(\lambda \right)\;\Phi (c_{0,\lambda }(1-e^{-\tau_{\lambda }})\\ & + & c_{1,\lambda }(1-e^{-m\;\tau^{L}})+c_{2,\lambda }(e^{m\;\tau^{L}}-1)) \end{array}$$ (52)

where *τ*_λ_ = *k*_λ_ *L* = *E*_λ_ *CL* and $\tau^{L}=\bar{k}L={\bar{E}}^{L}CL$ are the spectral and mean extinction optical thicknesses, respectively (see Section 2.1 and Fig 2), *p*^*i*^(λ) is the incident spectrum (see Section 2.1 and Fig 3), $m=\sqrt{3(1-\Phi )}$, $b=\frac{1+2/3\;m}{1-2/3\;m}$, *d*_λ_ = *τ*_λ_/(*mτ*^*L*^) and

$$\begin{array}{ccc} c_{0,\lambda } & = & \frac{1}{1-d_{\lambda }^{\;2}}\\ c_{1,\lambda } & = & -c_{0,\lambda }\;d_{\lambda }\;\frac{e^{-(\tau_{\lambda }+m\;\tau^{L})}\left(1-2/3\;d_{\lambda }\;m\right)-b\;\left(1+2/3\;d_{\lambda }\;m\right)}{e^{-2\;m\;\tau^{L}}\left(1-2/3\;m\right)-b\;\left(1+2/3\;m\right)}\\ c_{2,\lambda } & = & -c_{1,\lambda }\;b-c_{0,\lambda }\;d_{\lambda }\;\frac{1+2/3\;d_{\lambda }\;m}{1-2/3\;m} \end{array}$$

The results presented in Table 2 indicate that this approximation does not improve accuracy either significantly or systematically compared to zero-order approximation (see $\Delta (\bar{\text{P1}})$). For TATA^+^, which is accurately described by zero-order approximation and the gray model, relative errors are comparable: $|\Delta \left(\bar{\text{P1}}\right)|≃|\Delta \left(j=0\right)|≃|\Delta \left(\text{gray}\right)|$. For Eosin Y, accuracy is slightly improved, but the relative error exceeds 10% as soon as $\tau_{S}^{L}>0.6$. For Rhodamine B, the relative error is even greater compared to zero-order expansion only.

The key point here is that the $\bar{\text{P1}}$ approximation gives results that approach those provided by the gray equivalent model presented in Section 3.3 when scattering optical thickness increases ($\Delta \left(\bar{\text{P1}}\right)\to \Delta \left(\text{gray}\right)$ as $\tau_{S}^{L}\to \infty$). This is due to the fact that the angular distribution of intensity comes closer to the functional form assumed by the P1 approximation in Eq 48, due to isotropic scattering. As a result, multiple scattering is well described as soon as $\tau_{S}^{L}≳0.5$ and $\Delta \left(\bar{\text{P1}}\right)≃\Delta \left(\text{gray}\right)$ in this case. But on the other hand, the higher the $\tau_{S}^{L}$, the greater the inelastic scattering effects, which are neglected in the gray model at the root of the $\bar{\text{P1}}$ approximation (see Section 3.3). Overall, the $\bar{\text{P1}}$ approximation never finds its niche in the present case study. However, we kept this approximation because it would provide accurate results for photosensitizers with small spectral variations over the luminescence spectral range, since the gray model would be accurate in that case (see Section 3.3). We cannot demonstrate this statement with the typical photosensitizers that we selected because extinction cross section has large spectral variations over the luminescence spectral range. However, we can demonstrate it in the following virtual case: we use the same properties as Rhodamine B in Fig 2, except that the extinction cross section is gray and equal to ${\bar{E}}^{L}=24943$ m^2^.mol^−1^ (see Table 1). Comparing the Monte Carlo reference solution and the $\bar{\text{P1}}$ solution for $\tau_{A}^{i}=4.6$ we obtain $\Delta (\bar{\text{P1}})=0.01$, indicating a very good accuracy.

#### 4.2.2 P1 approximation applied to an elastic-scattering model for luminescence radiation

In order to improve the description of inelastic scattering effects that prevented the previous $\bar{\text{P1}}$ approximation from providing significant results, here we apply the P1 approximation to the elastic-scattering model presented in Section 3.3:

$$\begin{array}{c} P_{A}≃P_{A}^{\left(\hat{\text{P1}}\right)}=P_{A}^{\left(0\right)}+{\hat{P}}_{A}^{\left(S,P1\right)} \end{array}$$ (53)

where $P_{A}^{\left(0\right)}$ is the reference zero-order absorptance given in Eq 43 and ${\hat{P}}_{A}^{\left(S,P1\right)}$ is the luminescence absorptance resulting from both elastic and P1 approximations (see Eq 55).

An analytical expression of ${\hat{P}}_{A}^{\left(S,P1\right)}$ is obtained by following exactly the same steps as in Eqs 45 to 51 and S1 Appendix but working with the elastic RTE Eq 35 instead of the gray RTE Eq 31, and using the definition of absorptance given in Eq 24 instead of its gray version Eq 33:

$$\begin{array}{ccc} {\hat{P}}_{A}^{\left(S,P1\right)} & = & \int_{0}^{+\infty }d\lambda \;p^{i}\left(\lambda \right)\;\int_{0}^{+\infty }d\lambda^{\prime }\;p^{L}\left(\lambda^{\prime }\right)\;\Phi ({\hat{c}}_{0,\lambda ,\lambda^{\prime }}(1-e^{-\tau_{\lambda }})\\ & + & {\hat{c}}_{1,\lambda ,\lambda^{\prime }}(1-e^{-m\;\tau_{\lambda^{\prime }}})+{\hat{c}}_{2,\lambda ,\lambda^{\prime }}(e^{m\;\tau_{\lambda^{\prime }}}-1)) \end{array}$$ (54)

where *τ*_λ_ = *k*_λ_ *L* = *E*_λ_ *CL* is the extinction optical thickness (see Section 2.1 and Fig 2), *p*^*i*^(λ) and *p*^*L*^(λ) are the incident and luminescence spectrum respectively (see Section 2.1 and Fig 3), $m=\sqrt{3(1-\Phi )}$, $b=\frac{1+2/3\;m}{1-2/3\;m}$, *d*_λ,λ′_ = *τ*_λ_/(*mτ*_λ′_) and

$$\begin{array}{ccc} {\hat{c}}_{0,\lambda ,\lambda^{\prime }} & = & \frac{1}{1-d_{\lambda ,\lambda^{\prime }}^{\;2}}\\ {\hat{c}}_{1,\lambda ,\lambda^{\prime }} & = & -{\hat{c}}_{0,\lambda ,\lambda^{\prime }}\;d_{\lambda ,\lambda^{\prime }}\;\frac{e^{-(\tau_{\lambda }+m\;\tau_{\lambda^{\prime }})}\left(1-2/3\;d_{\lambda ,\lambda^{\prime }}\;m\right)-b\;\left(1+2/3\;d_{\lambda ,\lambda^{\prime }}\;m\right)}{e^{-2\;m\;\tau_{\lambda^{\prime }}}\left(1-2/3\;m\right)-b\;\left(1+2/3\;m\right)}\\ {\hat{c}}_{2,\lambda ,\lambda^{\prime }} & = & -{\hat{c}}_{1,\lambda ,\lambda^{\prime }}\;b-{\hat{c}}_{0,\lambda ,\lambda^{\prime }}\;d_{\lambda ,\lambda^{\prime }}\;\frac{1+2/3\;d_{\lambda ,\lambda^{\prime }}\;m}{1-2/3\;m} \end{array}$$

The relative error $\Delta (\hat{\text{P1}})$ obtained with this model is reported in Table 2. As expected from previous discussions (including Section 3.3), accuracy is significantly improved compared to $\bar{\text{P1}}$ approximation, in particular for optical thicknesses *τ*^*L*^ > 0.1 involving the strong impact of inelastic scattering. Double spectral integration in Eq 55 is the price to pay for this improvement. As a result, $\hat{\text{P1}}$ approximation is highly accurate for low-to-intermediate optical thicknesses, generating errors which are always below 2% for TATA^+^ and Eosin Y. Indeed, the two validity conditions for $\hat{\text{P1}}$ approximation are satisfied in these situations: first, the elastic-scattering model presented Section 3.3 is accurate (Δ(*elastic*) < 2%) and second, P1 approximation well describes multiple scattering in the framework of this elastic model ($\Delta \left(\hat{\text{P1}}\right)≃\Delta \left(\text{elastic}\right)$, see the discussion on P1 approximation validity in paragraphs below Eq 53). For Rhodamine B, on the other hand, the error increases by up to 26% because, even if the second condition is satisfied ($\Delta \left(\hat{\text{P1}}\right)≃\Delta \left(\text{elastic}\right)$ for *τ*^*L*^ > 1), the elastic-scattering model at the root of the $\hat{\text{P1}}$ approximation is not relevant (Δ(*elastic*) > 10%). This is due to high scattering optical thicknesses combined with significant inelastic effects, as discussed in Section 3.3.

### 4.3 Single-scattering approximation

The $\hat{\text{P1}}$ approximation developed in the previous section fails to describe absorptance for Rhodamine B photosensitizer, with $\Delta (\hat{\text{P1}})$ errors higher than the contribution of scattering orders *j* > 1 (see Table 3). Therefore, the description of luminescence radiation thanks to first-order scattering expansion is investigated here.

#### 4.3.1 Single-scattering approximation applied to the reference model

In the previous section, we were able to accurately account for multiple scattering, but important effects due to inelastic scattering were missing. Here we aim to describe inelastic scattering more accurately, but to do so we have to make a concession in the description of multiple scattering. In Section 3.2 we noticed that most of the luminescence contribution is due to photons absorbed after one scattering event only. Therefore, the expansion in successive orders of scattering is here truncated to the first order, which leads to a simple analytic expression [35, 50]:

$$\begin{array}{c} P_{A}≃P_{A}^{\left(j\leq 1\right)}=P_{A}^{\left(0\right)}+P_{A}^{\left(1\right)} \end{array}$$ (55)

where $P_{A}^{\left(0\right)}$ is the trivial zero-order absorptance given in Eq 43 and $P_{A}^{\left(1\right)}$ is given in Eq 58.

The expression of $I_{\lambda }^{\left(0\right)}$ given in Eq 41 is substituted in the RTE Eq 15 for first-order intensity $I_{\lambda }^{\left(1\right)}$, leading to the ordinary linear differential equation

$$\begin{array}{c} \mu \frac{dI_{\lambda }^{\left(1\right)}\left(x,\mu \right)}{dx}=-k_{\lambda }I_{\lambda }^{\left(1\right)}\left(x,\mu \right)+\int_{0}^{+\infty }d\lambda^{\prime }p^{L}\left(\lambda \right)\;\frac{\Phi k_{\lambda^{\prime }}}{2}q_{0,\lambda^{\prime }}\;e^{-k_{\lambda^{\prime }}x} \end{array}$$ (56)

whose solution is

$$\begin{array}{c} I_{\lambda }^{\left(1\right)}\left(x,\mu \right)=\{\begin{array}{cc} \int_{0}^{x}\frac{dx^{\prime }}{\mu }e^{-\frac{k_{\lambda }\left(x-x^{\prime }\right)}{\mu }}p^{L}\left(\lambda \right)\int_{0}^{+\infty }d\lambda^{\prime }\;\frac{\Phi k_{\lambda^{\prime }}}{2}q_{0,\lambda^{\prime }}\;e^{-k_{\lambda^{\prime }}x^{\prime }} & \text{for}\;\mu >0\\ \int_{x}^{L}\frac{dx^{\prime }}{-\mu }e^{-\frac{k_{\lambda }\left(x-x^{\prime }\right)}{\mu }}p^{L}\left(\lambda \right)\int_{0}^{+\infty }d\lambda^{\prime }\;\frac{\Phi k_{\lambda^{\prime }}}{2}q_{0,\lambda^{\prime }}\;e^{-k_{\lambda^{\prime }}x^{\prime }} & \text{for}\;\mu <0 \end{array} \end{array}$$ (57)

Substituting this expression in Eq 24 and performing symbolic integration leads to (see details in S2 Appendix):

$$\begin{array}{ccc} P_{A}^{\left(1\right)} & = & \int_{0}^{+\infty }d\lambda \;p^{i}\left(\lambda \right)\;\int_{0}^{+\infty }d\lambda^{\prime }\;p^{L}\left(\lambda^{\prime }\right)\;\frac{\tau_{\lambda^{\prime }}\;\Phi \left(1-\Phi \right)}{2}\{\frac{1}{\tau_{\lambda }}[Ei\left(-\tau_{\lambda^{\prime }}\right)\left(1+e^{-\tau_{\lambda }}\right)\\ & - & Ei\left(-\tau_{\lambda^{\prime }}-\tau_{\lambda }\right)+\frac{1}{2}\;\ln(\frac{\tau_{\lambda }+\tau_{\lambda^{\prime }}}{\tau_{\lambda^{\prime }}}{)}^{2}+e^{-\tau_{\lambda }}(\frac{1}{2}\;\ln(\frac{\tau_{\lambda }-\tau_{\lambda^{\prime }}}{\tau_{\lambda^{\prime }}}{)}^{2}-Ei\left(\tau_{\lambda }-\tau_{\lambda^{\prime }}\right))]\\ & + & \left(e^{-\tau_{\lambda }}-1\right)(Ei\left(-\tau_{\lambda^{\prime }}\right)+\frac{1}{\tau_{\lambda^{\prime }}}\left(e^{-\tau_{\lambda^{\prime }}}-1\right))\} \end{array}$$ (58)

where $Ei=\int_{-\infty }^{x}\frac{e^{t}}{t}\;dt$ is the exponential integral function (which is available in most scientific computing tools and applications).

Note that applying the single-scattering approximation to the reference model or to the elastic-scattering model presented in Section 3.3 leads to the exact same expression. Indeed, these two models lead to identical zero- and first-order terms in the scattering expansion (higher-order terms are different) because our elastic-scattering model includes spectral redistribution when ballistic photons move from population (0) to population (1). Thus the numerical evaluation of Eq 58 requires double integration, just as for the $\hat{\text{P1}}$ approximation in Eq 55.

The relative error Δ(*j* ≤ 1) recorded with this model is reported in Table 2. Note that Δ(*j* ≤ 1) = −*P*^(*j*>1)^ in Table 3 and that first-order scattering expansion was already discussed in Section 3.2.

Single-scattering approximation leads to an error below or equal to 5% for TATA^+^ and Eosin Y, for every tested optical thickness. It is slightly less accurate than $\hat{\text{P1}}$ approximation, but still a valuable approximation. At the contrary, as expected, accuracy for Rhodamine B is better with single-scattering approximation than with $\hat{\text{P1}}$ approximation. Nevertheless, Rhodamine B is definitely challenging: the relative error Δ(*j* ≤ 1) rapidly exceeds 10% when absorption optical thickness increases. Overall, single-scattering approximation is fairly accurate when *τ*^*L*^ < 1, which corresponds to a significant improvement compared to zero-order scattering approximation.

#### 4.3.2 Single-scattering approximation applied to the gray model for luminescence radiation

The above approximation provides valuable results but requires double numerical integration. Here, the gray approximation presented in Section 3.3 is applied to the first-order term, in order to eliminate spectral integration over the luminescence spectrum in $P_{A}^{\left(1\right)}$ (see Eq 58): $P_{A}^{\left(1\right)}≃{\bar{P}}_{A}^{\left(1\right)}$. In this way we obtain an expression for the single-scattering approximation $P_{A}^{\left(\bar{\text{SS}}\right)}$ that is even more straightforward to evaluate:

$$\begin{array}{c} P_{A}≃P_{A}^{\left(\bar{\text{SS}}\right)}=P_{A}^{\left(0\right)}+{\bar{P}}_{A}^{\left(1\right)} \end{array}$$ (59)

where $P_{A}^{\left(0\right)}$ is given in Eq 43 and ${\bar{P}}_{A}^{\left(1\right)}$ is given in Eq 62.

The same gray equivalent medium representation as in Section 3.3 is applied to Eq 15 with *j* = 1, leading to the following RTE for first-order intensity ${\bar{I}}_{\lambda }^{\left(1\right)}≃\int_{0}^{+\infty }d\lambda \;I_{\lambda }^{\left(1\right)}$:

$$\begin{array}{c} \mu \frac{d{\bar{I}}^{\left(1\right)}}{dx}=-\bar{k}{\bar{I}}^{\left(1\right)}\left(x,\mu \right)+\int_{0}^{+\infty }d\lambda^{\prime }\;\frac{\Phi k_{\lambda^{\prime }}}{2}\int_{-1}^{1}d\mu^{\prime }\;I_{\lambda^{\prime }}^{\left(0\right)}\left(x,\mu^{\prime }\right) \end{array}$$ (60)

with the boundary condition

$$\begin{array}{c} {\bar{I}}_{\lambda }^{\left(1\right)}\left(x,\mu \right)=0 \end{array}$$ (61)

Then the same approach as in Eqs 56 to 58 and S2 Appendix leads to

$$\begin{array}{ccc} {\bar{P}}_{A}^{\left(1\right)} & = & \int_{0}^{+\infty }d\lambda \;p^{i}\left(\lambda \right)\;\frac{\tau^{L}\Phi \left(1-\Phi \right)}{2}\{\frac{1}{\tau_{\lambda }}[Ei\left(-\tau^{L}\right)\left(1+e^{-\tau_{\lambda }}\right)-Ei\left(-\tau^{L}-\tau_{\lambda }\right)\\ & + & \frac{1}{2}\;\ln(\frac{\tau_{\lambda }+\tau^{L}}{\tau^{L}}{)}^{2}+e^{-\tau_{\lambda }}(\frac{1}{2}\;\ln(\frac{\tau_{\lambda }-\tau^{L}}{\tau^{L}}{)}^{2}-Ei\left(\tau_{\lambda }-\tau^{L}\right))]\\ & + & \left(e^{-\tau_{\lambda }}-1\right)(Ei\left(-\tau^{L}\right)+\frac{1}{\tau^{L}}\left(e^{-\tau^{L}}-1\right))\} \end{array}$$ (62)

where $\tau^{L}=\bar{k}L={\bar{E}}^{L}CL$ is the mean extinction optical thickness (see Section 2.1), and other definitions are given below Eq 58.

The results in Table 2 present the relative error $\Delta (\bar{\text{SS}})$ obtained with this model. Surprisingly, it provides better accuracy than the previous model, even though inelastic spectral effects are here neglected: $|\Delta \left(\bar{\text{SS}}\right)|<|\Delta \left(j\leq 1\right)|$. The results are remarkably accurate in every tested configuration, with a relative error below 5% (except Rhodamine B for $\tau_{S}^{L}=0.952$ where $\Delta (\bar{\text{SS}})=6.2\%$).

This remarkable accuracy results in fact from the compensation of two errors:

- On the one hand, truncating scattering expansion always underestimates absorptance, as discussed in Section 2.3. The magnitude of this underestimation is provided by Δ(*j* ≤ 1) in Table 2.
- On the other hand, gray approximation for luminescence radiation always overestimates absorptance (concavity property of the integrand), as discussed in Section 3.3.

Therefore, even if this approximation is of great interest for the study of photosensitized and photocatalytic systems, we cannot be sure that these two antagonistic effects always have similar absolute values and compensate for each other. For example, if a photosensitizer were studied with a gray extinction cross section on the luminescence spectral range (see Section 3.3), then $\Delta \left(\bar{\text{SS}}\right)=\Delta \left(j\leq 1\right)$ and accuracy would be significantly degraded at high optical thicknesses. Such situations should rather be addressed using the $\bar{\text{P1}}$ approximation, which would be accurate when $\tau_{S}^{L}>1$ (see Section 4.2).

To conclude, the use of this gray single-scattering approximation requires some precautions, but provides accurate results for every typical case investigated in this paper.

## 5 Practical implementation for overall quantum yield estimation

In a previous publication [18], the overall quantum yield of bio-inspired H_2_ production implemented in a benchmark photoreactor was estimated without taking into account luminescence. Hereafter we revisit these results and discuss the effects of luminescence on quantum yield measurements. In particular, the method established in the previous sections will be implemented in order to identify a relevant analytical approximate solution based on optical thickness analysis.

**Fig 4:**
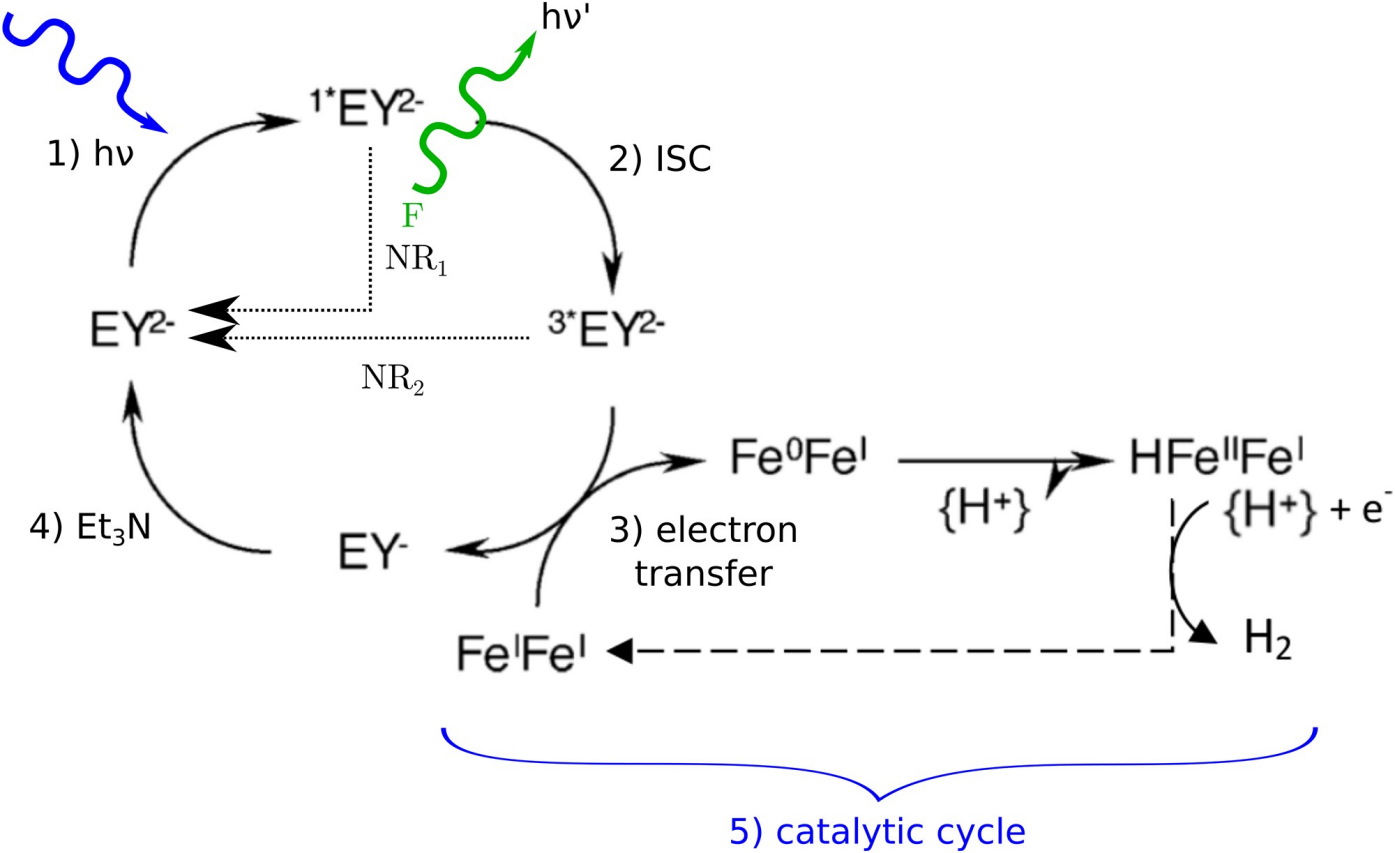
Proposed mechanism. Hydrogen production with the fluorescent photocatalytic system in homogeneous phase [21, 51, 52].

The photocatalytic system is an aqueous solution of an diiron-thiolate (FeFe) complex used as a proton reduction catalyst, fluorescent Eosin Y (EY^2−^) as the photosensitizer and triethylamine (Et_3_N) as a sacrificial electron donor. Such a photoreactive system can operate in water with the mechanism (see Fig 4) proposed as follows [21]:

1. When EY^2−^ absorbs photon energy, electrons are excited to a higher electronic level before rapidly relaxing to the first electronic state (the singlet ^1*^EY^2−^),
2. In the most favorable case for hydrogen production, electrons go through a spin conversion by intersystem crossing (ISC) into a triplet state (^3*^EY^2−^),
3. Next, an electron is transferred to the diiron catalyst,
4. To close the Eosin Y cycle, EY^−^ produced by the oxydoreduction reaction between ^3*^EY^2−^ and catalyst, EY^2−^ is regenerated by an electron transfer from Et_3_N,
5. Finally, a succession of oxidation-reduction and protonation with the catalyst, extensively described and analyzed, arises to produce hydrogen [51, 52].

Mechanisms unfavorable for hydrogen production (called loss mechanisms) can be described as follow:

- ^1*^EY^2−^ can relax by non-radiative emission (see the NR_1_ mechanism in Fig 4), thermalization or quenching. These phenomena cause a reduction in singlet state concentration.
- Assuming ^1*^EY^2−^ is converted into ^3*^EY^2−^ by ISC, relaxing to the ground state by phosphorescence does not occur at 25°C [53], but non-radiative emission (see the NR_2_ mechanism in Fig 4) still exists, resulting in a reduction in the triplet state concentration.
- Photons absorbed by the catalyst do not lead to hydrogen production. Therefore, the study of this specific catalyst implies taking into account a second absorbing species in the models. This will be presented in Section 5.2.

**Fig 5:**
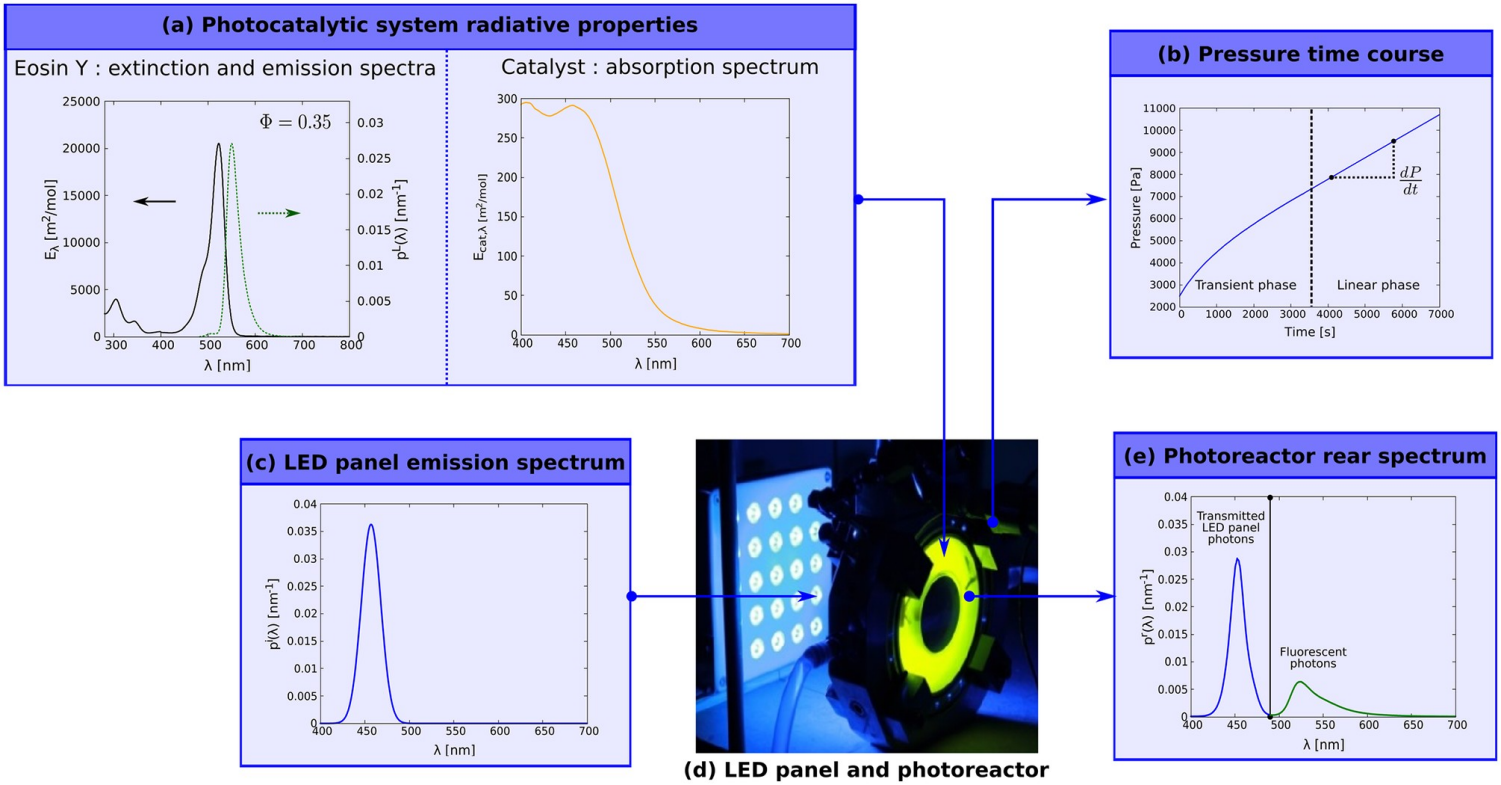
Materials. **(a)** Photocatalytic system radiative properties—left: Eosin Y (24 *μ*M) UV-visible molar extinction cross-section *E*_λ_, fluorescence emission spectrum *p*^*L*^(λ) and quantum yield Φ = 0.35 in a mixture of triethylamine (10% vol.) in water at pH 10.5 [18, 21]; right: Catalyst (1.66 mM) molar absorption cross section in methanol. UV-visible spectra were obtain using a Shimadzu UV-visible UV-160 A spectrophotometer and emission spectra from a Shimadzu RF-1501 spectrofluorimeter [18]—**(b)** Example of experimental pressure time course presenting transient and linear regimes with the slope $\frac{dP}{dt}$ in the linear regime—**(c)** wavelength probability density function incident source *i.e*. LED panel emission spectrum *p*^*i*^(λ)—**(d)** photograph of the photoreactor containing the fluorescent photocatalytic system illuminated by the blue LED panel—**(e)** Example of the wavelength probability density function spectrum *p*^*r*^(λ) of photons measured at the rear of the reactor composed of transmitted LED panel photons and fluorescent photons.

The singlet state of Eosin Y ^1*^EY^2−^ can drop to the ground state by fluorescence emission (see the F mechanism in Fig 4), and this was observed as shown in Fig 5e. As previously discussed, this mechanism cannot simply be described as a loss mechanism at the spatial scale of a photoreactor, since for Eosin Y an overlap exists between molar extinction cross section and emission spectrum. This means that photons emitted by fluorescence at one location, with a wavelength belonging to the overlapping, can be absorbed by Eosin Y at another location and potentially participate in the hydrogen production mechanism [54].

### 5.1 Materials and methods

#### 5.1.1 Photocatalytic system preparation

The catalyst synthesis and the photocatalytic system solution preparation protocols are detailed in [18, 21].

#### 5.1.2 Radiative properties

Eosin Y UV-visible extinction cross section *E*_λ_ and catalyst UV-visible absorption cross section *E*_*cat*,λ_ spectra were measured with a UV-160A Shimadzu spectrophotometer (see Fig 5a) [18]. The Eosin Y fluorescent emission spectrum was measured using a Shimadzu RF-1501 spectrofluorimeter and is presented in Fig 5a. The Eosin Y overall fluorescence quantum yield Φ = 0.35 was identified by minimising the mean squared errors on transmittance between measurements and Monte Carlo algorithm results [54].

#### 5.1.3 Photoreactor and incident light source

The reaction was implemented in a laboratory-scale photoreactor extensively described and characterized elsewhere [18, 19, 55]. The gas tight photoreactor of 190 mL is a square-section torus of thickness L = 2.5 cm with two translucent glass faces at the front and the rear (see Fig 5d). It was filled with a liquid volume *V*_*L*_ = 160 mL; consequently the headspace gas volume *V*_*G*_ was equal to 30 mL. The liquid photocatalytic system was composed of fluorescent Eosin Y at concentration *C* and the catalyst at concentration *C*_*cat*_, in a mixture of triethylamine (10% vol.) and water [18]. Eosin Y and the catalyst absorb radiation (see Fig 5a). The photoreactor was perfectly mixed and the temperature was controlled at 25.0 ± 0.1°C by water circulation in the photoreactor vessel jacket [55].

Incident radiation was obtained from a 25-LED panel positioned at a distance of 15 cm from the reactor, providing quasi-collimated blue light with a spectrum *p*^*i*^(λ) centered at a wavelength of 457 nm (see Fig 5c). The photon flux density entering the medium at the front face of the photoreactor, accurately controlled and easily modifiable, was measured via actinometry experiments, as described in [19].

In such a configuration, the radiative transfer theory can be approximated as a one-dimensional problem.

#### 5.1.4 Spectral light flux density measurements at the rear of the photoreactor

To carry out the radiative analysis, we measured the mean photon flux density exiting the reactor *q*_*r*_ measured at the rear glass window using a LI-COR quantum sensor (LI-190) connected to a LI-189 portable LI-COR Quantum meter/Radiometer/Photometer. This mean photon flux density at the rear of the photoreactor was obtained by measuring the photon flux density at 10 different positions. However, this device counts only the total hemispherical photon flux density and was not able to determine spectral distribution. Thus our experimental setup was complemented with a spectrometer (Ocean Optics USB 2000+) equipped with an optical fiber (QP 400–2-SR) and a cosine corrector (Ocean Optics CC-3) coupled to the fiber. The spectrometer was linked via USB connection to a computer running Oceanview software. Spectral distribution at the rear of the reactor *p*^*r*^(λ) was thus obtained (see Fig 5e).

#### 5.1.5 Hydrogen reaction rate measurement

Hydrogen was produced in the gas tight reactor under irradiation; this was indicated by an increase in pressure (see Fig 5b) measured using a pressure sensor (Keller PA 33X) located in the headspace of the reactor. To analyze this phenomenon, a complete mass analysis of the hydrogen was performed, taking into account a transient regime followed by a linear regime. During the linear regime, the mean volumetric rate of hydrogen production $\langle r_{H_{2}}\rangle$ can be calculated from the linear variation in pressure *P* with time *t* on the basis of the slope $\frac{dP}{dt}$ [55]. A complete analysis of the gas phase was routinely performed with a micro gas chromatograph to demonstrate that only hydrogen is produced.

#### 5.1.6 A typical experiment

For a given photocatalytic system (Eosin Y and catalyst concentrations), the incident photon flux density *q*_0_ in front of the photoreactor is fixed. The photon flux exiting at the rear of the photoreactor as well as the rear emission spectrum and the pressure time course are measured. These measurements are repeated for different *q*_0_ values. This experiment is implemented for different Eosin Y and catalyst concentrations.

### 5.2 Results

We demonstrated that the photocatalytic system follows a linear thermokinetic coupling law [18]. This means that a plot of the hydrogen production rate $\langle r_{H_{2}}\rangle$ *versus* MVRPA ${\langle A\rangle }^{\left(•\right)}$ is a straight line, the slope of which being the overall quantum yield *φ*^(•)^ (see Eq 2):

$$\begin{array}{c} \varphi^{\left(•\right)}=\frac{\left\langle r_{H_{2}}\right\rangle }{{\left\langle A\right\rangle }^{\left(•\right)}} \end{array}$$ (63)

From the experimental hydrogen production rate $\langle r_{H_{2}}\rangle$, the overall quantum yield is identified by Eq 63 according to several methods used to obtain MVRPA, which are:

#### 5.2.1 Measure neglecting fluorescence (notation (•)≡ (exp, 0))

We published in [18] experimental MVRPA results that were used to estimate *φ*^(exp,0)^. Those results were obtained from the difference between the incident photon flux density entering the front of the reactor *q*_0,λ_ = *q*_0_ *p*^*i*^(λ) and the measured hemispherical photon flux density exiting the reactor at the rear. Photon flux at the rear of the photoreactor is composed of transmitted ballistic (blue) photons generated by the LED panel in spectral interval λ∈ [400 nm; 490 nm] and scattered (fluorescent) photons emitted by Eosin Y in interval λ∈ [490 nm; 630 nm], as presented in Fig 5e. The fact that these spectral intervals do not overlap allows us to distinguish between ballistic and fluorescence radiation at the rear to obtain the radiative balance on ballistic radiation only:

$$\begin{array}{c} {\left\langle A\right\rangle }^{\left(\text{exp},0\right)}=\frac{1}{L}\int_{400\;nm}^{490\;nm}d\lambda \;\left(q_{0,\lambda }-q_{r,\lambda }\right)\;f_{\lambda } \end{array}$$ (64)

where we introduced a factor *f*_λ_ to account for catalyst absorption (see Eq 65).

Estimating the total MVRPA including fluorescence radiation would require measuring the outward hemispherical photon flux density exiting the front of the photoreactor. Indeed, ballistic radiation exits the medium at the rear only but fluorescence exits at the rear and front faces. However our device does not allow such measurements, and with luminescence the calculation of MVRPA from models is required.

#### 5.2.2 Calculation of MVRPA from models

MVRPA is calculated with Eq 23, from the following absorptance models that have been presented in previous sections:

- Neglecting fluorescence (see Section 4): notation (•) ≡ (Φ = 0)
- Reference model taking fluorescence into account (see Section 2.5): notation where superscript (•) is dropped
- Gray Single-scattering analytical approximation (see Section 4.3): notation $\left(•\right)\equiv (\bar{\text{SS}})$

Absorption by the catalyst is simply added to the models established in previous sections by modifying the radiative properties as follows:

- *k*_λ_ → *k*_λ_ + *k*_*cat*,λ_ with the extinction coefficient of the catalyst *k*_*cat*,λ_ = *C*_*cat*_ *E*_*cat*,λ_
-
$\Phi \to \alpha_{s,\lambda }=\Phi \frac{k_{\lambda }}{k_{\lambda }+k_{cat,\lambda }}$

In addition, the calculation of MVRPA used for the formulation of thermokinetic coupling should not include the radiation absorbed by the catalyst since it does not lead to hydrogen production. To perform this calculation the integrand in Eq 24 (and the weight of the Monte Carlo algorithm) are multiplied by the probability *f*_λ_ that a photon absorbed is absorbed by Eosin Y molecules (rather than by the catalyst):

$$\begin{array}{c} f_{\lambda }=\frac{k_{\lambda }\left(1-\Phi \right)}{k_{\lambda }\left(1-\Phi \right)+k_{cat,\lambda }} \end{array}$$ (65)

where Φ is equal to 0 when luminescence is neglected. Note that, integrated over the spectral range from 400 nm to 490 nm, factor *f* varies between 79% and 96% for the concentration range studied (see Table 4). Indeed, absorption by the catalyst is unfavorable for hydrogen production and should remain low.

**Table 4:** Overall quantum yields of the homogeneous photoreactive system at different catalyst (*C*_*cat*_) and Eosin Y (*C*) concentrations in mol.m^−3^. Results obtained from Eq 63, using several methods to estimate 〈A〉: *φ*^(exp,0)^ uses measurements on ballistic radiation (fluorescence is neglected), *φ* uses the reference Monte Carlo calculation, *φ*^(Φ=0)^ uses model results when fluorescence is neglected, $\varphi^{\left(\bar{\text{SS}}\right)}$ uses the gray single-scattering analytical approximation presented in Section 4.3. Relative error *δ* with respect to the reference value *φ* is provided (see Eq 66). Optical thicknesses $\tau_{A}^{i}$ and $\tau_{S}^{L}$ are calculated from Eqs 4 and 6, with ${\bar{E}}^{i}=2556\;m^{2}.mol^{-1}$ for the LED emission spectrum.

			Reference	Neglecting luminescence		Approximation
*C*_*cat*_	*C*	$\tau_{A}^{i}\;|\;\tau_{S}^{L}$	*φ*	*φ*^(exp,0)^	*φ*^(Φ=0)^ | *δ*(Φ = 0)	$\varphi^{\left(\bar{SS}\right)}\;|\;\delta \left(\bar{\text{SS}}\right)$
0.075	0.05	2.22 | 1.19	0.76 ± 0.08	0.61 ± 0.08	0.62 ± 0.08 | -20%	0.74 ± 0.08 | -3%
0.1	0.2	8.87 | 4.77	0.47 ± 0.04	0.44 ± 0.04	0.41 ± 0.04 | -13%	0.48 ± 0.04 | 2%
0.1	0.45	19.95 | 10.74	0.25 ± 0.02	0.22 ± 0.02	0.22 ± 0.02 | -12%	0.26 ± 0.02 | 4%

The resulting formulation of absorptance when accounting for catalyst absorption is provided in S3 Appendix.

Reflection and refraction at the rear of the photoreactor are not included in the models, since the transmission of the water / glass / air interface is ≃ 90% for our spectral range of work (value obtained from both measurements and geometrical optics calculations). Moreover, less than 10% of the incident radiation reaches the rear glass for the absorption optical thicknesses studied in Table 4 ($e^{-\tau_{A}^{i}}<0.1$). Therefore, only 1% of the incident radiation is reflected back into the medium.

#### 5.2.3 Results gathered in Table 4 also include

- Absorption and scattering optical thicknesses defined in Eqs 4 and 6, with ${\bar{E}}^{i}=2556\;m^{2}.mol^{-1}$ for the LED emission spectrum (${\bar{E}}^{L}=2728\;m^{2}.mol^{-1}$ is unchanged, see Table 1). Absorption by the catalyst has very little impact on photon transport analysis and is therefore not included in these optical thickness calculations.
- The relative difference
  $$\begin{array}{c} \delta \left(X\right)=\frac{\varphi^{\left(X\right)}-\varphi }{\varphi } \end{array}$$ (66)
  between the reference overall quantum yield *φ* (obtained with the reference MVRPA given by the Monte Carlo method) and the overall quantum yields *φ*^(*X*)^ obtained from the MVRPA models denoted (X) above.

### 5.3 Discussion

The overall quantum yield *φ* in Table 4 is expected to vary as a function of Eosin Y concentration. The increase in *φ* when Eosin Y concentration decreases could be explained by quenching phenomena: collisions between Eosin Y excited states and other molecules (quenchers) such as Et_3_N, H_2_O, or Eosin Y itself, for instance, limits the singlet and triplet state yield required for hydrogen production [18]. This effect is out of the scope of this article and we focus rather on the difference between *φ* values obtained using different MVRPA estimation methods.

#### 5.3.1 Comparison between measurements and model

First, we compare the overall quantum yields obtained when luminescence is neglected, using both experimental MVRPA measurements in *φ*^(exp,0)^ and model results in *φ*^(Φ=0)^. The results in Table 4 show no significant differences, validating the radiative properties of the model.

#### 5.3.2 Overall fluorescence effect

Based on the results presented in Section 3, the analysis of optical thicknesses enables us to anticipate the error *δ*(Φ = 0) observed when fluorescence is neglected:

- For the scattering optical thicknesses $\tau_{S}^{L}>1$ investigated in Table 4, we expect significant effects of fluorescence, leading to absorptance overestimation. According to Eq 66, we therefore expect the overall quantum yield *φ* to be underestimated when fluorescence is neglected (*φ* is inversely proportional to absorptance). Indeed, we verify that *δ*(Φ = 0) < 0 in Table 4.
- The first row in Table 4 addresses a configuration where $\tau_{A}^{i}=2.22$ and $\tau_{S}^{L}=1.19$, that is intermediate between the third and fourth columns in Table 2, for which we recorded errors Δ(Φ = 0) in MVRPA estimation equal to 29.5% and 20.5% respectively. We therefore expect *δ*(Φ = 0) ≃ 25% in that configuration, which is confirmed.
- The second and third rows in Table 4 address situations with higher optical thicknesses. As discussed in Section 3.1, the error Δ(Φ = 0) on MVRPA decreases as the absorption optical thickness $\tau_{A}^{i}$ increases, and indeed the error |*δ*(Φ = 0)| decreases in Table 4. However, *δ*(Φ = 0) values in the second and third rows are very close, despite the fact that optical thicknesses are twice as high in the third row. Indeed, we observe that the error on MVRPA decreases, without approaching zero since fluorescence radiation cannot be completely absorbed, even for very high concentrations. Here, we are approaching the asymptote.

These results confirm that fluorescence must be taken into account to accurately estimate the overall quantum yield; otherwise we are subject to a relative error between 12% and 20% for the experimental cases presented in this paper.

#### 5.3.3 Gray single-scattering analytical approximation

Finally, in order to easily take fluorescence into account when estimating the overall quantum yield (without using an advanced numerical method such as Monte Carlo), we developed convenient analytical approximation solutions in Section 4. The validity of these approximations depends on the scattering optical thickness $\tau_{S}^{L}$ (see the summary in Conclusions and perspectives). The gray single-scattering approximation $\bar{\text{SS}}$ presented in Section 4.3 is the most relevant here, since $\tau_{S}^{L}>1$ and spectral variations of Eosin Y are important. This approximation is remarkably accurate, leading to a relative error $\delta (\bar{\text{SS}})$ for the overall quantum yield estimation of less than 4% in every tested situation.

## 6 Conclusions and perspectives

We have presented a radiative model for luminescence radiation which can be used to study any photosensitized reactive system for photoreactor engineering applications. The analysis of luminescence radiation transport is approached from the multiple inelastic-scattering point of view, using Monte Carlo simulations, successive orders of scattering expansion formalism and five physical approximations that are combined to provide five convenient analytical approximate solutions. The study of four photosensitizers that are representative of photocatalytic systems for solar fuel production by artificial photosynthesis leads us to the following conclusions (see Table 2):

- the proportion of incident radiation that is absorbed within photoreactive media is reduced in the presence of luminescence because 1) part of the incident radiation is backscattered and leaves the medium and 2) part of the spectral distribution is shifted to a non-absorbing spectral range;
- these effects are substantial and cannot be totally neglected when studying photoreactors;
- the zero-order scattering approximation in Eq 43 is a relevant approximation that is straightforward to implement and that improves the description of photon transport considerably compared to simply neglecting luminescence. The gray single-scattering approximation $\bar{\text{SS}}$ in Eq 59 is more accurate: it estimates absorptance with a relative error below 6% in every tested situation. In addition, it only requires the exponential integral function to be computed (available in most scientific computing tools and applications);
- when absorption optical thickness $\tau_{A}^{i}$ is adjusted such that incident radiation is absorbed as much as possible and converted into solar fuels or valuable products, scattering optical thickness $\tau_{S}^{L}$ often exceeds 0.1 (see Eq 6). In this case, two major conceptual and practical difficulties must be tackled: inelastic scattering and multiple scattering;
- complex effects of inelastic scattering are due to non-gray extinction cross section *E*_λ_ in the luminescence spectral range $\left[\lambda_{min}^{L},\lambda_{max}^{L}\right]$ (see Fig 2): the greater the spectral variations and $\tau_{S}^{L}$, the more difficult these effects are to grasp;
- of course, the effect of multiple scattering increases with optical thickness: the contribution of scattering orders *j* decreases with *j*, and the higher $\tau_{S}^{L}$ is, the larger the contribution of high scattering orders becomes. We record significant contributions up to *j* = 3 in the cases investigated;
- the following analytical approximate solutions can be used to describe photon absorption with an error below 5%:
  - *zero-order scattering approximation* in Eq 43 when $\tau_{S}^{L}<0.5$; it provides accurate results for $\text{Ru}{\left[\text{Bpy}\right]}_{3}^{2+}$ and TATA^+^, representing half of the test cases investigated in this paper,
  - *inelastic single-scattering approximation* in Eq 55 and $\hat{\text{P1}}$ *approximation* in Eq 53 when $\tau_{S}^{L}<1$ and spectral variations in *E*_λ_ in $\left[\lambda_{min}^{L},\lambda_{max}^{L}\right]$ are significant; they provide accurate results for $\text{Ru}{\left[\text{Bpy}\right]}_{3}^{2+}$, TATA^+^ and Eosin Y, but require double numerical integration;
  - *gray* $\bar{\text{P1}}$ *approximation* in Eq 44 when spectral variations in *E*_λ_ in $\left[\lambda_{min}^{L},\lambda_{max}^{L}\right]$ are small (almost gray, which is unlikely) and $\tau_{S}^{L}>0.5$; none of the studied photosensitizers corresponds to this case;
  - *gray single-scattering approximation* $\bar{\text{SS}}$ in Eq 59 when spectral variations in *E*_λ_ in $\left[\lambda_{min}^{L},\lambda_{max}^{L}\right]$ are large, whatever $\tau_{S}^{L}$; it provides remarkably accurate results in every tested configuration, but this accuracy arises from the compensation of two errors. Therefore, even if this approximation is of great interest for the study of photocatalytic systems, its use requires some precautions.
- Cases with high scattering optical thickness combined with strong spectral variations in *E*_λ_ in $\left[\lambda_{min}^{L},\lambda_{max}^{L}\right]$, as for Rhodamine B, can hardly be treated with confidence in any way that does not involve advanced numerical methods such as Monte Carlo. Even if the $\bar{\text{SS}}$ approximation provides surprisingly accurate results for Rhodamine B, we cannot ensure *a priori* that this is the case for *every* photosensitizer.

The work presented in this article also allows us to take another look at overall quantum yield estimation. The overall quantum yield of a photoreactive system composed of Eosin Y as the photosensitizer and a bio-inspired catalyst for H_2_ production was initially obtained without accounting for the fluorescence of Eosin Y in the treatment of experimental results [18]. We revisited these results here and show that:

- neglecting fluorescence on absorptance clearly affects the results of overall quantum yields, which are underestimated since absorptance is overestimated,
- according to the optical thickness of the experimental configuration, the gray single-scattering analytical approximation was selected. It provides results with a error of less than 4% on overall quantum yield estimation, enabling fluorescence to be taken into account without having to implement an advanced numerical method such as Monte Carlo.

Some interesting perspectives can also be drawn up. First of all, this work could be extended to the evaluation of the mean volumetric rate of photon absorption 〈A〉, and to the estimation of transmittance in a 1D Cartesian system (photoreactors or spectrophotometer cuvette). This should not require too much additional work, since all the formal work presented in this article is performed at local intensity, from which it is easy to evaluate transmittances (cosine-weighted integration over the outgoing hemisphere) instead of absorptance. For the same reasons, the assessment of the local rate of photon absorption can be implemented without major difficulty: the final integration over reaction volume is simply suppressed in the formulations of absorptance presented in this paper. This is necessary when dealing with a non-linear thermokinetic coupling law [16, 50]. This work could also be extended by considering reflection at the interfaces at both sides of the slab or using uncollimated incident radiation. An extension of the 1D algorithm toward any complex 3D geometry for photoreactive applications is also at hand thanks to the computational tools for ray tracing in complex geometry that have been developed over the last twenty years by the computer graphics research community in the Monte Carlo framework [47, 56].

Finally, many of the tools presented and discussed in this work could find interesting applications for the quantitative use of Fast Fluorescence Rate (FFR) or Pulse Amplitude Modulation (PAM) fluorescence measurements using microalgae in the field of photobioreactor engineering [57], as well as remote sensing [58, 59]. To do so, elastic scattering by particles will have to be included in the models.

## Supporting information

S1 Appendix P1 approximation. (ZIP)

S2 Appendix Single-scattering approximation. (ZIP)

S3 Appendix Monte Carlo algorithm and extended models with catalyst absorption. (ZIP)

S1 Nomenclature (PDF)
