# Supplementary material for: Radiative analysis of luminescence in photoreactive systems: Application to photosensitizers for solar fuel production
Source: PLoS One. 2021 Jul 22;16(7):e0255002. doi: 10.1371/journal.pone.0255002 (PMC8297781; doi:10.1371/journal.pone.0255002)
Supplement: S1 Appendix — (ZIP) [file pone.0255002.s001.zip › S1_Appendix.pdf]

Supporting information : S1 Appendix. P1 approximation

Caroline SUPPLIS, Jérémi DAUCHET, Victor GATTEPAILLE, Fabrice GROS, Thomas VOURC'H, Jean-François CORNET.

Solution of Eq 50 is

$$\bar{G}^{(S,P1)}(x) = C_0\, e^{\bar{k}\, m\, x} + C_1\, e^{-\bar{k}\, m\, x} + \int_0^{+\infty} d\lambda\, \frac{3\,\Phi\, k_\lambda/\bar{k}}{m^2 - \left(k_\lambda/\bar{k}\right)^2}\, q_{0,\lambda}\, e^{-k_\lambda x} \tag{S1-1}$$

with $m = \sqrt{3(1-\Phi)}$. For convenience we introduce spectral coefficients $C_{0,\lambda}$ and $C_{1,\lambda}$ satifying $C_{0,1} = \int_0^{+\infty} d\lambda\, C_{i,\lambda}$ and write Eq S1-1 as $\bar{G}^{(S,P1)}(x) = \int_0^{+\infty} d\lambda\, f_\lambda(x)$ with

$$f_\lambda(x) = C_{0,\lambda}\, e^{\bar{k}\, m\, x} + C_{1,\lambda}\, e^{-\bar{k}\, m\, x} + \frac{3\,\Phi\, k_\lambda/\bar{k}}{m^2 - \left(k_\lambda/\bar{k}\right)^2}\, q_{0,\lambda}\, e^{-k_\lambda x} \tag{S1-2}$$

Note that $f_\lambda(x)$ cannot be interpreted as a spectral distribution for $\bar{G}^{(S,P1)}$: luminescence radiation is gray in the framework of the present approximation. Thus, based on the Marshak boundary conditions in Eq 51, we prefer to work with

$$f_\lambda(0) - \frac{2}{3\,\bar{k}}\frac{d\,f_\lambda}{dx}(0) = 0 \tag{S1-3}$$

$$f_\lambda(L) + \frac{2}{3\,\bar{k}}\frac{d\,f_\lambda}{dx}(L) = 0$$

to determine coefficients $C_{i,\lambda}$ in Eq S1-2, instead of C_i in Eq S1-1. This is more restrictive than the original problem but it gives a more convenient expression which only requires one spectral integration over the incident spectrum (see Eq 52):

$$C_{0,\lambda} = -C_{1,\lambda}\, b - \frac{3\,\Phi\, k_\lambda/\bar{k}}{m^2 - \left(k_\lambda/\bar{k}\right)^2}\, \frac{1 + 2/3\, k_\lambda/\bar{k}}{1 - 2/3\, m} \tag{S1-4}$$

$$C_{1,\lambda} = -\frac{3\,\Phi\, k_\lambda/\bar{k}}{m^2 - \left(k_\lambda/\bar{k}\right)^2}\, \frac{(1 - 2/3\, k_\lambda)\, e^{-(k_\lambda + \bar{k}\, m)L} - b\,(1 + 2/3\, k_\lambda)}{(1 - 2/3\, \bar{k}\, m)\, e^{-2\bar{k}\, m\, L} - b\,(1 + 2/3\, \bar{k}\, m)} \tag{S1-5}$$

where $b = \frac{1+2/3\, m}{1-2/3\, m}$.

Finally, the luminescence absorptance in Eq 33 is expressed as a function of irradiance:

$$\bar{\mathcal{P}}_A^{(S,P1)} = \frac{1}{q_0}\int_0^L dx\,(1-\Phi)\,\bar{k}\,\bar{G}^{(S,P1)}(x) \tag{S1-6}$$

leading to the final expression in Eq 52.
