# Supplementary material for: Radiative analysis of luminescence in photoreactive systems: Application to photosensitizers for solar fuel production
Source: PLoS One. 2021 Jul 22;16(7):e0255002. doi: 10.1371/journal.pone.0255002 (PMC8297781; doi:10.1371/journal.pone.0255002)
Supplement: S2 Appendix — (ZIP) [file pone.0255002.s002.zip › S2_Appendix.pdf]

Supporting information : S2 Appendix. Single-scattering approximation

Caroline SUPPLIS, Jérémi DAUCHET, Victor GATTEPAILLE, Fabrice GROS, Thomas VOURC'H, Jean-François CORNET.

In this appendix we will focus on obtaining the absorptance single-scattering analytical expression presented by Eq 58. First, the irradiance solution from Eq 57 integrated over directions and wavelengths is presented:

$$\begin{aligned} G^{(1)}(x) = &\int_0^{+\infty} d\lambda\, p^L(\lambda) \int_0^{+\infty} d\lambda'\, p^i(\lambda') \\ &\left(\int_0^1 d\mu \int_0^x \frac{dx'}{\mu} e^{-\frac{k_\lambda (x-x')}{\mu}} \frac{\Phi k_{\lambda'}}{2} q_0\, e^{-k_{\lambda'} x'} \right. \\ &\left. + \int_{-1}^0 d\mu \int_x^L \frac{dx'}{-\mu} e^{-\frac{k_\lambda (x-x')}{\mu}} \frac{\Phi k_{\lambda'}}{2} q_0\, e^{-k_{\lambda'} x'} \right) \end{aligned} \tag{S2-1}$$

where we used the definition of the incident spectrum $p^i(\lambda') = \frac{q_{0,\lambda'}}{q_0}$. In the following, dummy integration variables λ and λ' are interchanged and integration over wavelengths is set aside for convenience; we will concentrate on the function $G^{(1)}_{\lambda,\lambda'}(x)$ such that :

$$G^{(1)}(x) = \int_0^{+\infty} d\lambda'\, p^L(\lambda') \int_0^{+\infty} d\lambda\, p^i(\lambda)\, G^{(1)}_{\lambda,\lambda'}(x) \tag{S2-2}$$

where

$$\begin{aligned} G^{(1)}_{\lambda,\lambda'}(x) &= q_0 \int_0^x dx' e^{-k_\lambda x'} k_\lambda \frac{\Phi}{2} \int_0^1 \frac{d\mu}{-\mu} e^{-\frac{k_{\lambda'}(x-x')}{\mu}} \\ &+ q_0 \int_x^E dx' e^{-k_\lambda x'} k_\lambda \frac{\Phi}{2} \int_{-1}^0 \frac{d\mu}{\mu} e^{-\frac{k_{\lambda'}(x-x')}{\mu}} \\ &= G^{(1)+}_{\lambda,\lambda'}(x) + G^{(1)-}_{\lambda,\lambda'}(x) \end{aligned} \tag{S2-3}$$

with the functions $G^{(1)+}_{\lambda,\lambda'}(x)$ and $G^{(1)-}_{\lambda,\lambda'}(x)$ that will be solved separately hereafter.

- **Analytical solution for $G^{(1)+}_{\lambda,\lambda'}(x)$:**
  Integration over directions is resolved

$$G^{(1)+}_{\lambda,\lambda'}(x) = q_0 k_\lambda \frac{\Phi}{2} \int_0^x dx' e^{-k_\lambda x'} \left[-Ei\left(-\frac{k_{\lambda'}(x-x')}{\mu} \right) \right]_0^1 \tag{S2-4}$$

Since $\lim_{x\to+\infty} Ei(-x) = 0$, the integration over locations x that must be solved is:

$$G^{(1)+}_{\lambda,\lambda'}(x) = q_0 k_\lambda \frac{\Phi}{2} \int_0^x -dx' e^{-k_\lambda x'} Ei\left(-k_{\lambda'}(x-x')\right) \tag{S2-5}$$

leading to the following expression:

$$\begin{aligned} G^{(1)+}_{\lambda,\lambda'}(x) = q_0 \frac{\Phi}{2} \Big[&e^{-k_\lambda x'} Ei(-\bar{k}(x-x')) \\ &- e^{-k_\lambda x} Ei((k_\lambda - k_{\lambda'})(x-x')) \Big]_0^x \end{aligned} \tag{S2-6}$$

then, by taking the limit expression when $x' \longmapsto x$ for the upper integration limit, we obtain the final solution for $G^{(1)+}_{\lambda,\lambda'}(x)$:

$$G^{(1)+}_{\lambda,\lambda'}(x) = q_0 \frac{\Phi}{2} \left\{ e^{-k_\lambda x} \left(\frac{1}{2} ln \left(\frac{k^2_{\lambda'}}{(k_{\lambda'} - k_\lambda)^2} \right) + Ei((k_\lambda - k_{\lambda'})x) \right) - Ei(-k_{\lambda'} x) \right\} \quad \text{(S2-7)}$$

- **Analytical solution for $G^{(1)-}_{\lambda,\lambda'}(x)$:**
  Integration over direction is resolved

$$G^{(1)-}_{\lambda,\lambda'}(x) = q_0 k_\lambda \frac{\Phi}{2} \int_x^L dx' e^{-k_\lambda x'} \left[Ei\left(-\frac{k_{\lambda'}(x - x')}{\mu} \right) \right]_{-1}^{0} \quad \text{(S2-8)}$$

Since $\lim_{x \to +\infty} Ei(-x) = 0$, the integration over locations x to solve is:

$$G^{(1)-}_{\lambda,\lambda'}(x) = q_0 k_\lambda \frac{\Phi}{2} \int_x^L -dx' e^{-k_\lambda x'} Ei\left(-k_{\lambda'}(x - x')\right) \quad \text{(S2-9)}$$

leading to the following expression:

$$G^{(1)-}_{\lambda,\lambda'}(x) = q_0 \frac{\Phi}{2} \left[e^{-k_\lambda x'} Ei(-k_{\lambda'}(x - x')) - e^{-k_\lambda x} Ei((k_\lambda - k_{\lambda'})(x - x')) \right]_x^L \quad \text{(S2-10)}$$

taking the limit expression when $x' \longmapsto x$ for the lower integration limit, we obtain the final solution for $G^{(1)-}_{\lambda,\lambda'}(x)$

$$G^{(1)-}_{\lambda,\lambda'}(x) = q_0 \frac{\Phi}{2} \left\{ e^{-k_\lambda L} Ei(k_{\lambda'}(x - L)) - e^{-k_\lambda x} Ei((k_\lambda + k_{\lambda'})(x - L)) - e^{-k_{\lambda'} x} \frac{1}{2} ln \left(\frac{k^2_{\lambda'}}{(k_\lambda + k_{\lambda'})^2} \right) \right\} \quad \text{(S2-11)}$$

- **Irradiance of the first scattering order:**
  Finally, irradiance of the first scattering order in Eq S2-2 is written with:

$$G^{(1)}_{\lambda,\lambda'}(x) = q_0 \frac{\Phi}{2} \left[e^{-k_\lambda x} \left\{ \frac{1}{2} ln \left(\frac{(k_\lambda + k_{\lambda'})^2}{(k_{\lambda'} - k_\lambda)^2} \right) - Ei((k_\lambda + k_{\lambda'})(x - L)) + Ei((k_\lambda - k_{\lambda'})x) \right\} e^{-k_\lambda L} Ei(k_{\lambda'}(x - L)) - Ei(-k_{\lambda'} x) \right] \quad \text{(S2-12)}$$

- **First-order absorptance:**
  According to Eqs. 24 and S2-2

$$\mathcal{P}^{(1)}_A = \frac{L}{q_0} \int_0^{+\infty} d\lambda' \, p^L(\lambda') \int_0^{+\infty} d\lambda \, p^i(\lambda) \int_0^L dx (1 - \Phi) k_{\lambda'} G^{(1)}_{\lambda,\lambda'}(x) \quad \text{(S2-13)}$$

We introduce the function:

$$\mathcal{P}^{(1)}_{A,\lambda,\lambda'} = \frac{L}{q_0} \int_0^L dx (1 - \Phi) k_{\lambda'} G^{(1)}_{\lambda,\lambda'}(x) \quad \text{(S2-14)}$$

such that:

$$\mathcal{P}^{(1)}_A = \int_0^{+\infty} d\lambda \, p^i(\lambda) \int_0^{+\infty} d\lambda' p^L(\lambda') \, \mathcal{P}^{(1)}_{A,\lambda,\lambda'} \quad \text{(S2-15)}$$

To obtain the expression of the absorptance first scattering order, $\mathcal{P}^{(1)}_{A,\lambda,\lambda'}$ is solved hereafter.

Let us replace Eq S2-12 in Eq S2-14 and separate the expression into several terms to simplify the solution process:

• Term 1:

$$\text{Term 1} = ... \frac{1}{2}\, ln\left(\frac{(k_\lambda + k_{\lambda'})^2}{(k_{\lambda'} - k_\lambda)^2}\right)\int_0^L dx\, e^{-k_\lambda x} \;..... \tag{S2-16}$$

integration over locations does not present any difficulties, and leads to the analytical solution

$$\text{Term 1} = \frac{1}{2k_\lambda} ln\left(\frac{(k_\lambda + k_{\lambda'})^2}{(\bar{k} - k_\lambda)^2}\right)[1 - e^{k_\lambda L}] \tag{S2-17}$$

• Term 2:

$$\text{Term 2} = ... \int_0^L dx\, e^{-k_\lambda x} Ei\left((k_\lambda + k_{\lambda'})(x - L)\right) \;.... \tag{S2-18}$$

and the primitive function for term 2 is:

$$\text{Term 2} = \frac{1}{k_\lambda}\left[e^{-k_\lambda x} Ei\left((k_\lambda + k_{\lambda'})(x - L)\right) - e^{-k_\lambda L} Ei(k_{\lambda'}(x - L))\right]_0^L \tag{S2-19}$$

searching for the limit for $x \longmapsto L$ for the upper integration limit leads to the following analytical solution:

$$\begin{aligned}\text{Term 2} = \frac{1}{k_\lambda}\Bigg[&e^{-k_\lambda x}\{\frac{1}{2} ln\left(\frac{(k_\lambda + k_{\lambda'})^2}{k_{\lambda'}^2}\right) + Ei(-k_{\lambda'} L)\} \\ &- Ei(-(k_\lambda + k_{\lambda'})L)]\end{aligned} \tag{S2-20}$$

• Term 3:

The expression to be solved is:

$$\text{Term 3} = ... \int_0^L dx\, e^{-k_\lambda x} Ei\left((k_\lambda - k_{\lambda'})x\right) \;.... \tag{S2-21}$$

which leads to

$$\text{Term 3} = \frac{1}{k_\lambda}\left[Ei(-k_{\lambda'} x) - e^{-k_\lambda x} Ei((k_\lambda - k_{\lambda'})x)\right]_0^L \tag{S2-22}$$

and taking the limit for $x \longmapsto 0$ for the lower integration limit gives the following expression:

$$\begin{aligned}\text{Term 3} = \frac{1}{k_\lambda}\Big[&Ei(-k_{\lambda'} L) - e^{-k_\lambda L} Ei((k_\lambda - k_{\lambda'})L) \\ &- \frac{1}{2} ln\left(\frac{k_{\lambda'}^2}{(k_{\lambda'} - k_\lambda)^2}\right)\Big]\end{aligned} \tag{S2-23}$$

• Term 4:

$$\text{Term 4} = ... \; e^{-k_\lambda L}\int_0^L dx\, Ei(k_{\lambda'}(x - L)) \;.... \tag{S2-24}$$

Integration over locations leads to:

$$\text{Term 4} = e^{-k_\lambda L}\left[(x - L) Ei(k_{\lambda'}(x - L)) - \frac{1}{k_{\lambda'}} e^{k_{\lambda'}(x-L)}\right]_0^L \tag{S2-25}$$

to finally obtain the analytical solution for term 4 by taking the limit for $x \longmapsto L$ for the upper integration limit:

$$\text{Term 4} = e^{-k_\lambda L}\left[-\frac{1}{k_{\lambda'}} + LEi(-k_{\lambda'}L) + \frac{1}{k_{\lambda'}}e^{-k_{\lambda'}L}\right] \tag{S2-26}$$

- Term 5:
  For the last term of Eq S2-14 the following expression must be solved

$$\text{Term 5} = ... \int_0^L dx\, Ei(-k_{\lambda'}x)\, ... \tag{S2-27}$$

This does not present any difficulties and leads to:

$$\text{Term 5} = LEi(-k_{\lambda'}L) + \frac{1}{k_{\lambda'}}e^{-k_{\lambda'}L} - \frac{1}{k_{\lambda'}} \tag{S2-28}$$

Finally, by gathering terms 1 to 5, the absorptance first scattering order $\mathcal{P}^{(1)}_{A,\lambda,\lambda'}$ is obtained:

$$\begin{aligned}\mathcal{P}^{(1)}_{A,\lambda,\lambda'} = \frac{k_{\lambda'}\Phi(1-\Phi)}{2}\Bigg\{\frac{1}{k_\lambda}\Big[&Ei((-k_{\lambda'}L)(1+e^{-k_\lambda L})) - Ei(-L(k_\lambda - k_{\lambda'})) \\ &+ \frac{1}{2}ln\left(\frac{(k_\lambda + k_{\lambda'})^2}{k^2_{\lambda'}}\right) + e^{-k_\lambda L}\left(\frac{1}{2}\, ln\left(\frac{(k_{\lambda'} - k_\lambda)^2}{k^2_{\lambda'}}\right) - Ei(L(k_\lambda - k_{\lambda'}))\right)\Big] \\ &+ (e^{-k_\lambda L} - 1)(LEi(-k_{\lambda'}L) + \frac{1}{k_{\lambda'}}(e^{-k_{\lambda'}L} - 1))\Bigg\}\end{aligned} \tag{S2-29}$$

Eq S2-29 is multiplied and divided by the slab thickness L to make the optical thicknesses appear, and integrated over the incident and luminescent spectra according to Eq S2-15 to obtain Eq 58.
