# Supplementary material for: Radiative analysis of luminescence in photoreactive systems: Application to photosensitizers for solar fuel production
Source: PLoS One. 2021 Jul 22;16(7):e0255002. doi: 10.1371/journal.pone.0255002 (PMC8297781; doi:10.1371/journal.pone.0255002)
Supplement: S3 Appendix — (ZIP) [file pone.0255002.s003.zip › S3_Appendix.pdf]

Supporting information : S3 Appendix. Monte Carlo algorithm and extended models with catalyst absorption

Caroline SUPPLIS, Jérémi DAUCHET, Victor GATTEPAILLE, Fabrice GROS, Thomas VOURC'H, Jean-François CORNET.

This appendix presents extended models and Monte Carlo algorithm we used to estimate absorptance with catalyst absorption :

- We record reference absorptance $\mathcal{P}_A$ from Monte Carlo algorithm detailed in section 1,
- Straightforward expression of absorptance neglecting fluorescence $\mathcal{P}_A^{(\Phi=0)}$ is presented in section 2,
- Absorptance calculated from gray single scattering analytical approximation $\mathcal{P}_A^{(\overline{\mathrm{SS}})}$ is developped in section 3

and the following rules were applied from expressions presented in the article :

- $k_\lambda \rightarrow k_\lambda + k_{cat,\lambda}$ with the extinction coefficient of the catalyst $k_{cat,\lambda} = C_{cat}E_{cat,\lambda}$
- $\Phi \rightarrow \alpha_{s,\lambda} = \Phi \frac{k_\lambda}{k_\lambda + k_{cat,\lambda}}$
- Monte Carlo algorithm as well as analytical absorptance expression are weighted by the probability f_λ :

$$f_\lambda = \frac{k_\lambda(1-\Phi)}{k_\lambda(1-\Phi)+k_{cat,\lambda}} \tag{S3-1}$$

1 Monte Carlo Algorithm

The Monte Carlo algorithm presented below was used to estimate the absorptance $\mathcal{P}_A$ for a given Eosin Y concentration C, catalytyst concentration C_{cat}, slab thickness L and radiative properties Φ, E_λ and $E_{cat,\lambda}$. It consists in sampling N independent realizations w_i, $i = 1, ...N$ with the following sampling procedure:

- **Step 1:** Initialization of Monte Carlo weight $w_i = 0$, emission location $\mathbf{r} = \mathbf{0}$ and emission direction $\mathbf{u} = \mathbf{e}_x$.
- **Step 2:** Wavelength λ is sampled according to the incident spectrum $p^i(\lambda)$, Eosin Y extinction coefficient $k_\lambda = C\,E_\lambda$ and catalyst absorption coefficient $k_{cat,\lambda} = C_{cat}\,E_{cat,\lambda}$ are interpolated from spectral database. The single scattering albedo $\alpha_{s,\lambda}$ is computed.
- **Step 3:** A first extinction length l is sampled over $[0, +\infty[$ according to the Bouguer extinction probability density function $p_L(l) = k_\lambda e^{-k_\lambda l}$ and the location is updated: $\mathbf{r} = \mathbf{r} + l\,\mathbf{u}$.
- **while** $0 \leq \mathbf{r}\cdot\mathbf{e}_x \leq L$ (location is inside the slab) **do**
  Step 4: Bernoulli test: uniform sampling of a realization ξ over $[0, 1]$

– **if** $\xi < \alpha_{s,\lambda}$ (scattering event) *then*
Step 4a.1: Scattering direction $\mathbf{u}$ is sampled according to the isotropic phase function.
Step 4a.2: Wavelength λ is sampled according to the luminescence spectrum $p^L(\lambda)$, Eosin Y extinction coefficient $k_\lambda = C\, E_\lambda$ and catalyst absorption coefficient $k_{cat,\lambda} = C_{cat}\, E_{cat,\lambda}$ are interpolated from spectral database. The single scattering albedo $\alpha_{s,\lambda}$ is computed.
Step 4a.3: Extinction length l is sampled over $[0, +\infty[$ according to the Bouguer extinction probability density function $p_L(l) = k_\lambda e^{-k_\lambda l}$ and the location is updated: $\mathbf{r} = \mathbf{r} + l\, \mathbf{u}$.
end

– **Else**
Step 4b.1: Path sampling is terminated due to absorption:
• weight is computed:
$w_i = f_\lambda$
• go to the end of Monte Carlo realization.
end

end

Absorptance is estimated as:

$$\mathcal{P}_A \simeq \frac{1}{N} \sum_{i=1}^{N} w_i \tag{S3-2}$$

with standard error:

$$\sigma(\mathcal{P}_A) = \frac{1}{\sqrt{N-1}} \sqrt{\frac{1}{N} \sum_{i=1}^{N} {w_i}^2 - \left(\frac{1}{N} \sum_{i=1}^{N} w_i \right)^2} \tag{S3-3}$$

2 Absorptance neglecting luminescence

Rules to take into account catalyst absorption are applied on Eq 40 leading to :

$$\mathcal{P}_A^{(\Phi=0)} = \int_0^{+\infty} d\lambda\; p^i(\lambda) f_\lambda\; (1 - e^{-(k_\lambda + k_{cat,\lambda})L}) \tag{S3-4}$$

3 Gray single-scattering approximation analytical solution

The expression for the gray single scattering approximation $\mathcal{P}_A \simeq \mathcal{P}_A^{(\overline{\mathrm{SS}})}$ (Eq 59) is :

$$\mathcal{P}_A \simeq \mathcal{P}_A^{(\overline{\mathrm{SS}})} = \mathcal{P}_A^{(0)} + \bar{\mathcal{P}}_A^{(1)} \tag{S3-5}$$

with

$$\mathcal{P}_A^{(0)} = \int_0^{+\infty} d\lambda\; (1 - \alpha_{s,\lambda}) f_\lambda\; p^i(\lambda)\; (1 - e^{-(k_\lambda + k_{cat,\lambda})L}) \tag{S3-6}$$

and

$$\bar{\mathcal{P}}_A^{(1)} = \int_0^{+\infty} d\lambda\, p^i(\lambda)\bar{f}(1-\bar{\alpha}_s)\frac{\alpha_{s,\lambda}(\bar{k}+\bar{k}_{cat})}{2}\left\{\frac{1}{k_\lambda + k_{cat,\lambda}}\left[Ei(-(\bar{k}+\bar{k}_{cat})L)(1+e^{-(k_\lambda+k_{cat,\lambda})L}))\right.\right.$$
$$- Ei(-((k_\lambda + k_{cat,\lambda}) - (\bar{k}+\bar{k}_{cat}))L)$$
$$+ \frac{1}{2}ln\left(\frac{((k_\lambda + k_{cat,\lambda}) + (\bar{k}+\bar{k}_{cat}))^2}{(\bar{k}+\bar{k}_{cat})^2}\right)$$
$$+ e^{-(k_\lambda+k_{cat,\lambda})L}\left\{\frac{1}{2}\ ln\left(\frac{((\bar{k}+\bar{k}_{cat}) - (k_\lambda + k_{cat,\lambda}))^2}{(\bar{k}+\bar{k}_{cat})^2}\right)\right.$$
$$\left.\left.-\ Ei(((k_\lambda + k_{cat,\lambda}) - (\bar{k}+\bar{k}_{cat}))L)\right\}\right]$$
$$\left. + (e^{-(k_\lambda+k_{cat,\lambda})L} - 1)(LEi(-(\bar{k}+\bar{k}_{cat})L) + \frac{1}{\bar{k}+\bar{k}_{cat}}(e^{-(\bar{k}+\bar{k}_{cat})L} - 1))\right\} \quad \text{(S3-7)}$$

where

- $\bar{k}_{cat} = \bar{E}_{cat}C_{cat}$ with $\bar{E}_{cat} = \int_0^{+\infty} d\lambda\, p^L(\lambda)E_{cat,\lambda} = 36\ \mathrm{m}^2.\mathrm{mol}^{-1}$ for the practical implementation example in Section 5.
- $\bar{\alpha}_s = \Phi\frac{\bar{k}}{\bar{k}+\bar{k}_{cat}}$
- $\bar{f} = \frac{\bar{k}(1-\Phi)}{\bar{k}(1-\Phi)+\bar{k}_{cat}}$
