# Supplementary material for: Radiative analysis of luminescence in photoreactive systems: Application to photosensitizers for solar fuel production
Source: PLoS One. 2021 Jul 22;16(7):e0255002. doi: 10.1371/journal.pone.0255002 (PMC8297781; doi:10.1371/journal.pone.0255002)
Supplement: S1 Nomenclature — (PDF) [file pone.0255002.s004.pdf]

Nomenclature

$\mathcal{A}(\mathbf{r})$ Local Volumetric Rate of Photons Absorbed (LVRPA) $[\mathrm{mol}_{h\nu}.\mathrm{m}^{-3}.\mathrm{s}^{-1}]$

$\langle\mathcal{A}\rangle$ Mean Volumetric Rate of Photons Absorbed (MVRPA) $[\mathrm{mol}_{h\nu}.\mathrm{m}^{-3}.\mathrm{s}^{-1}]$

A Absorbance [-]

C Concentration $[\mathrm{mol.m}^{-3}]$

$\mathbf{e}_x$ Unit vector along the x abscissa [-]

E Molar cross-section $[\mathrm{m}^2.\mathrm{mol}^{-1}]$

f Probability that a photon absorbed within the reactor is absorbed by the photosensitizer [-]

G Irradiance $[\mathrm{mol}_{h\nu}.\mathrm{m}^{-2}.\mathrm{s}^{-1}]$

H_{H_2} Henry constant of hydrogen in triethylamine and water $[\mathrm{m}^3.\mathrm{Pa.mol}^{-1}]$

I Intensity $[\mathrm{mol}_{h\nu}.\mathrm{m}^{-2}.\mathrm{sr}^{-1}.\mathrm{s}^{-1}]$

k Extinction coefficient $[\mathrm{m}^{-1}]$

l Extinction length [m]

L Slab thickness [m]

N Number of Monte Carlo algorithm iterations [-]

P Pressure [Pa]

$P^{(j)}$ Distribution of a scattering orders [-]

$p(\lambda)$ Spectral source probability density function $[\mathrm{nm}^{-1}]$

$p(\mu|\mu')$ Phase function $[\mathrm{sr}^{-1}]$

$\mathcal{P}_A$ Absorptance [-]

q_0 Photon flux density $[\mathrm{mol}_{h\nu}.\mathrm{m}^{-2}.\mathrm{s}^{-1}]$

R Gas constant $[\mathrm{J.K}^{-1}.\mathrm{mol}^{-1}]$

$\mathbf{r}$ Location ($\|\mathbf{r}\| =$ [m])

$r_i(\mathbf{r})$ Local volumetric chemical reaction rate $[\mathrm{mol}_i.\mathrm{m}^{-3}.\mathrm{s}^{-1}]$

$\langle r_i\rangle$ Mean volumetric chemical reaction rate $[\mathrm{mol}_i.\mathrm{m}^{-3}.\mathrm{s}^{-1}]$

t Time [s]

$\mathbf{u}$ Unit propagation direction [-]

V_L Liquid volume in the photoreactor [mL]

V_G Gas volume in the headspace of the photoreactor [mL]

w Monte Carlo algorithm weight [-]

$x = \mathbf{r}\cdot\mathbf{e}_x$ Abscissa in the slab [m]

Greek letters

α Single-scattering albedo with catalyst absorption [-]

δ Relative difference between overall quantum yields [-]

Δ Relative difference between absorptance models [-]

λ Radiation wavelength [m]

$\mu = \mathbf{u} \cdot \mathbf{e}_x$ Cosine of propagation direction [-]

σ Standard error [-]

τ Optical thickness [-]

Φ Luminescence quantum yield $\equiv$ single-scattering albedo [-]

φ Overall quantum yield [$\mathrm{mol}_i.\mathrm{mol}_{h\nu}^{-1}$]

Subscripts/Superscripts

A Refers to absorption

cat Refers to catalyst

λ Refers to wavelength

min Minimum value

max Maximum value

S Refers to scattering

$elastic$ Refers to elastic approximation

exp Refers to experimental measurement

$gray$ Refers to gray approximation

i Refers to incident source

j Refers to the jth order in the successive order of scattering expansion

L Refers to the luminescence source

$P1$ Relative to $P1$ approximation

r Relative to rear face of the photoreactor

S Refers to scattered (luminescence) radiation

SS Refers to Single-Scattering approximation

$\bar{}$ Refers to gray value

$\widehat{}$ Refers to elastic value

$1*$ Refers to singlet state

$3*$ Refers to triplet state

Acronyms

Et_3N	Triethylamine
EY^{2-}	Eosin Y molecule
F	Fluorescence
FeFe	Diiron thilate complex catalyst
ISC	Intersystem-crossing
MVRPA	Mean Volumetric Rate of Photons Absorbed
NR	Non Radiative
RTE	Radiative Transfer Equation
